# *Chondrochelia* Guţu, 2016 (Crustacea, Peracarida, Tanaidacea, Leptocheliidae) from North America: new species, redescription and distribution using morphological and molecular data

**DOI:** 10.7717/peerj.12773

**Published:** 2022-01-21

**Authors:** Jani Jarquín-González, Luis F. Carrera-Parra

**Affiliations:** 1 División de Estudios de Posgrado e Investigación, Instituto Tecnológico de Chetumal, Chetumal, Quintana Roo, México; 2 Departamento de Sistemática y Ecología Acuática, El Colegio de la Frontera Sur, Chetumal, Quintana Roo, México

**Keywords:** Taxonomy, Morphology, DNA barcoding, New species, SEM

## Abstract

Until now, four species of the genus *Chondrochelia* Guţu, 2016 have been recorded from America. Using morphological and molecular data, we were able to recognize and describe two new species, *Chondrochelia caribensis* sp. nov. from the Mexican Caribbean and *Chondrochelia winfieldi* sp. nov. from the Gulf of Mexico. We found significant genetic divergence values between species based on the nucleotide sequences of cytochrome oxidase subunit I to support the morphological data. Also, the range of distribution of two species: *Chondrochelia mexicana* (Jarquín-González, García-Madrigal & Carrera-Parra, 2015) and *Chondrochelia ortizi* (Jarquín-González, 2016), were expanded within their described geographic regions. In contrast, the supposed distribution of the Brazilian *C. dubia* in the Mexican Caribbean and the Gulf of Mexico was rejected. Additionally, *Chondrochelia algicola* (Harger, 1878) was redescribed based upon type material. Minute details and ornamentation of some structures of three species were examined using SEM.

## Introduction

Recently, Guţu (2016) established the leptocheliid genus *Chondrochelia* to include some species of *Leptochelia* Dana, 1849, most of them previously assigned to the “group *L. dubia*”. In America, only four species of *Chondrochelia* have been described, the first being *Chondrochelia dubia* (Krøyer, 1842), originally described within the genus *Tanais* Latreille, 1831, and based on a single female specimen. Later, Sars (1882) transferred this species to *Leptochelia*, while Sieg (1983) included at least 60 *Leptochelia* species as junior synonyms of *L. dubia*, consequently expanding its distribution worldwide. However, this expansive distribution should be considered doubtful because tanaidacean species have low natural dispersal capacity due to the lack of planktonic larvae and their generally tubicolous lifestyle (Bamber, 2010; Jakiel, Palero & Błażewicz, 2019). After an extensive taxonomic revision, Guţu (2016) transferred *L. dubia* to his new genus, *Chondrochelia*. For many years, it was considered that the male morphotype of *Chondrochelia savignyi* (Krøyer, 1842) from Madeira, Portugal was conspecific with *C. dubia* from Salvador Bahia, Brazil. However, this is incorrect and Bamber (2010), using an integrative taxonomic approach, showed that these species could no longer be considered synonymous.

*Chondrochelia algicola* (Harger, 1878) was the second species recognized in America. It was briefly described and illustrated as *Paratanais algicola* Harger, 1878 from Noank Harbor, Connecticut, USA. Later, Harger (1879) transferred it to *Leptochelia* based on the male’s morphology. Soon after this, Sars (1882) and Richardson (1905) synonymized *Leptochelia algicola* with *L. dubia* and *L. savignyi* respectively and for more than 100 years taxonomic authorities such as Lang (1973) and Sieg (1983) supported this synonymy. Nevertheless, according to Anderson (2013), *L. algicola* could be considered as a valid species because the synonyms proposed by Sieg (1983) have been in doubt. Recently, Guţu (2016) transferred *L. algicola* to *Chondrochelia*. However, a detailed description based on the type or topotypical material is needed to differentiate it from its supposed synonyms.

Jarquín-González, García-Madrigal & Carrera-Parra (2015) and Jarquín-González (2016) described the third and fourth species, *Chondrochelia mexicana* and *Chondrochelia ortizi*, from America. *Chondrochelia mexicana* was characterized by having a spiniform seta on article-2 of the male uropod endopod, while *C. ortizi* was distinguished by the form of the processes on the fixed finger of the male cheliped. Prior this study, *C. mexicana* was only known from Guerrero, Mexico (Mexican Pacific) and *C. ortizi* in the Gulf of Guanahacabibes and Isla de la Juventud, Cuba.

Herein, based on morphological and molecular analysis, we describe two new species, from the Mexican Caribbean and Gulf of Mexico. Specimens of both species were previously recorded as “*Leptochelia dubia*” (*e.g*. Suárez-Morales et al., 2004; García-Madrigal, Heard & Suárez-Morales, 2005). Also, the distribution of two described species: *Chondrochelia mexicana* and *Chondrochelia ortizi*, are expanded within their biogeographic regions, while the supposed presence of *C. dubia* in the Mexican Caribbean and the Gulf of Mexico is rejected. Furthermore, *Chondrochelia algicola* is redescribed based upon type materials and topotypical materials; as a result, we disagree with its proposed synonyms, and consider it to be a valid species.

## Materials and Methods

We analyzed the syntype specimens of *Paratanais algicola* (currently *Chondrochelia algicola*) deposited in the collections of the National Museum of the Natural History, Smithsonian Institution, Washington, USA (USNM). Other specimens studied were obtained from material deposited in the following collections: Acuario Nacional de Cuba (ANC), La Habana, Cuba; Marine Invertebrates of Universidad del Mar (UMAR), Puerto Ángel, Oaxaca, México; National Collection of Crustaceans (CNCR) of Universidad Nacional Autónoma de México; and the Reference Collection of Benthos (ECOSUR), El Colegio de la Frontera Sur, Chetumal, México.

The specimens were examined under a Carl Zeiss SV6 stereomicroscope. The total body length of specimens was measured from the anterior end of the cephalothorax (*i.e*., rostrum) to the posterior margin of the pleotelson. To determine the proportions of the morphological structures (*e.g*., antennules, pereopods) the total length was measured *vs* the width at the mid-length. Apart from mouthparts, dissections were performed on the right side of the body and the appendages were mounted in glycerol and sealed with transparent nail varnish. Drawings of the taxonomic structures were made using a camera lucida at 4×–40× magnification. The terminology used for anatomical features is based on Larsen (2003) and Guţu (2016). For the scanning electron microscopy (SEM) analysis, the specimens were dehydrated in a series of different concentration of hexamethyldisilazane (HMDS). Once air-dried, they were mounted on aluminum stubs and coated with gold for observation using a JEOL JSM-6010Plus-LA SEM at the Scanning Electron Microscopy Laboratory (LMEB), ECOSUR, Chetumal. Photographs of some diagnostic characters were obtained to complement the descriptions of the species.

For molecular studies the gene Cytochrome oxidase subunit I (COI) was used as it has been efficient in recognizing crustacean taxa, including peracarids (Larsen, 2001; Costa et al., 2007; Jarquín-González & Carrera-Parra, 2019; Błażewicz et al., 2019). The DNA was extracted using the whole organism following the protocol of Ivanova, deWaard & Hebert, 2006 and DNA barcoding was carried out at the Canadian Center for DNA Barcoding (University of Guelph), following the standard protocols of the program “Barcode of the Life.” Cytochrome oxidase subunit I (COI) nucleotide sequences were amplified by PCR using ZplankF1_t1 (5′-TGTAAAACGACGGCCAGTTCTASWAATCATAARGATATTGG-3′) and ZplankR1_t1 (5′-CAGGAAACAGCTATGACTTCAGGRTGRCCRAARAATCA-3′) primer set with the thermocycler program (94 °C for 40 s, 45 °C for 40 s, 72 °C for 1 min), then 35 cycles of (94 °C for 40 s, 51 °C for 40 s, 72 °C for 1 min) and a final extension of 72 °C for 5 min (Prosser, Martínez-Arce & Elías-Gutierrez, 2013). M13F (5′-TGTAAAA CGACGGCCAGT-3′) and M13R (5′-CAGGAAACAGCTATGAC-3′) primers (Messing, 1983) were used for sequencing. Sequence data, electropherograms, trace files, primer details, photographs, life stages, and collection localities for specimens are available within the project “*Leptochelia* from Mexico” at Barcode of Life Data System. Also, for the molecular analysis, some sequences of *Chondrochelia africana* (Larsen & Froufe, 2013), *C. dubia* (Krøyer, 1842), *C. savignyi* (Krøyer, 1842), *Leptochelia forresti* (Stebbing, 1896), and *L. longichelipes* (Lang, 1973) were obtained from the GenBank data base (Table 1).

**Table 1:** Specimens included in molecular analyses.

Species	Locality	BOLD System # process ID	GenBank accession no.	References
*C. caribensis* sp. nov.	Xcacel, Quintana Roo (Mexican Caribbean)	TANAI040-15 TANAI041-15		Current work
*C. winfieldi* sp. nov.	Isla Verde, Veracruz (Gulf of Mexico)	TANAI270-15 TANAI271-15 TANAI272-15 TANAI273-15 TANAI274-15		Current work
*C. ortizi*	Champotón, Campeche (Gulf of Mexico)	TANAI019-15		Current work
	Pinar del Río (Cuba)	TANAI089-15		
*C. mexicana*	La Boquilla, Oaxaca (Mexican Pacifc)	TANAI223-15 TANAI238-15 TANAI241-15 TANAI243-15		
	Manzanillo, Guerrero (Mexican Pacifc)	TANAI263-15 TANAI264-15 TANAI267-15		Current work
*C. dubia*	Fort Lauderdale, FL, USA		HM016215	Drumm (2010)
	Bijagos		JX316005	Larsen & Froufe (2013)
*C. africana*	Archipelago (Guinea Bissau)		JX316006	
*C. savignyi*	Phare de Saint-Mathieu (France)		JX402115	Larsen, Nagaoka & Froufe (2012)
	Arinaga beach, Gran Canarias		JX402117	
*L. forresti*	Dania Beach, Fl, USA		HM016206	Drumm (2010)
*L. longichelipes*	Belize		HM016201	Drumm (2010)

The sequences were aligned using ClustalW method, and no indels or stop codons were found. The final aligned dataset for analysis was 418 base pairs long. The selection of the best model substitution was determined according with the lowest Bayesian Information Criterion (BIC) score. As result, we used Hasegawa-Kishino-Yano (HKY), using a discrete Gamma distribution (+G) with five rate categories and by assuming that a certain fraction of sites is evolutionarily invariable (+I) as model to construct a tree using the Maximum Likelihood analysis. Additionally, we used the Kimura 2-Parameter (K2P) model to estimate the average evolutionary divergence over sequence pairs within and between species. All analyzes were carried out with the MEGA7 program (Kumar, Stecher & Tamura, 2015).

The electronic version of this article in portable document format will represent a published work according to the International Commission on Zoological Nomenclature (ICZN), and hence the new names contained in the electronic version are effectively published under that Code from the electronic edition alone. This published work and the nomenclatural acts it contains have been registered in ZooBank, the online registration system for the ICZN. The ZooBank LSIDs (Life Science Identifiers) can be resolved and the associated information viewed through any standard web browser by appending the LSID to the prefix http://zoobank.org/. The LSID for this publication is: urn:lsid:zoobank.org:pub:DB236268-1523-41AF-8701-16A3E1D696F0. The online version of this work is archived and available from the following digital repositories: PeerJ, PubMed Central, and CLOCKSS.

### Systematics

Superorder Peracarida Calman, 1904

Order Tanaidacea Dana, 1849

Suborder Tanaidomorpha Sieg, 1980

Family Leptocheliidae Lang, 1973

Subfamily Leptocheliinae Lang, 1973

**Genus *Chondrochelia* Guţu, 2016**

### *Chondrochelia algicola* (Harger, 1878)

Figures 1–6

**Figure 1:**
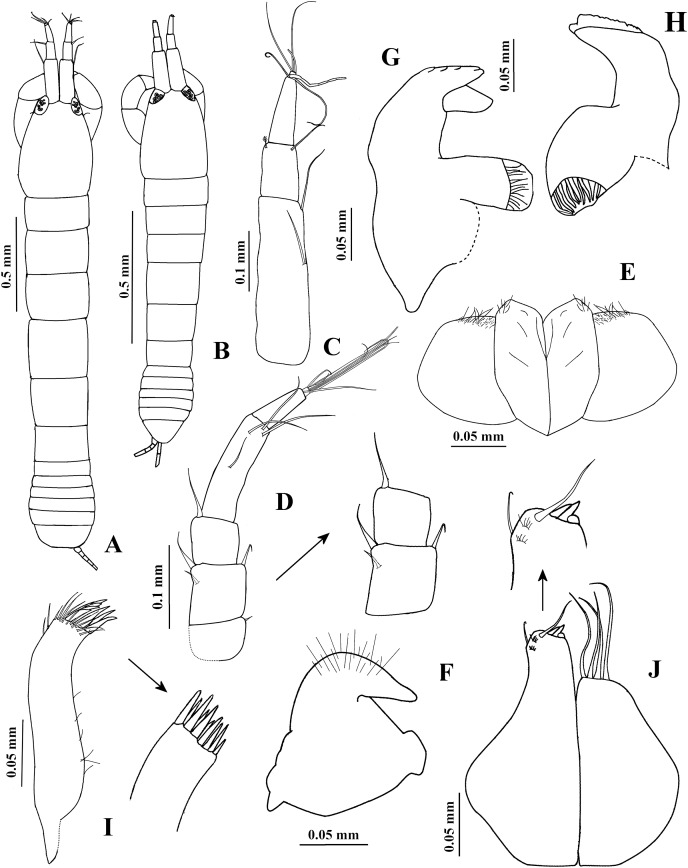
*Chondrochelia algicola*, type and topotype specimens. Syntype USNM 35963, ovigerous female, 2.4 mm. (A) Habitus. (C) Antennule. (D) Antenna. (E) Labium. (F) Labrum. (G) Left mandible. (H) Right mandible. (I) Maxillule. (J) Basis of maxilliped and endite. Topotype USNM 35964, juvenile female, 1.5 mm. (B) Habitus. Drawing credit: Jani Jarquín-González.

**Figure 2:**
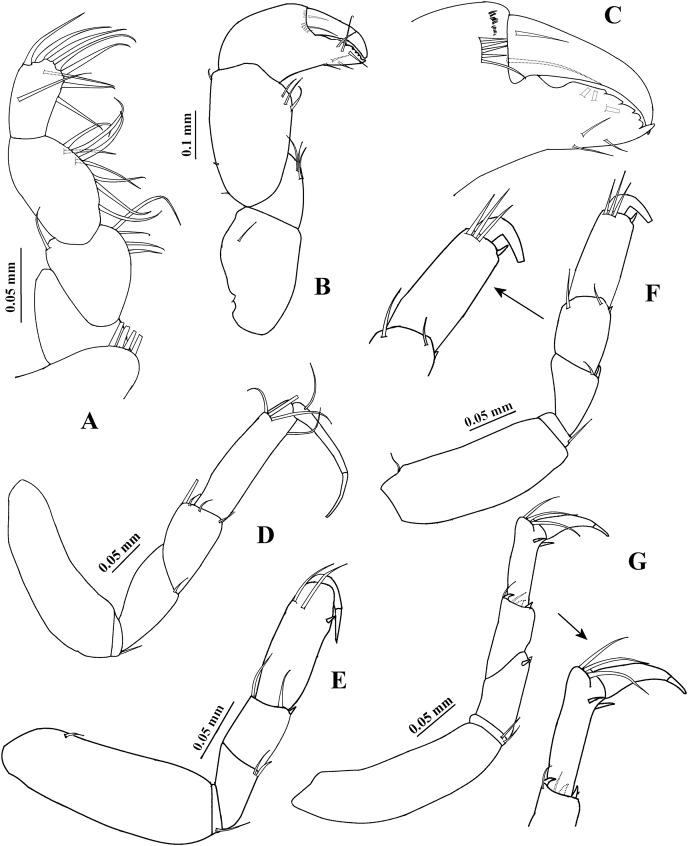
*Chondrochelia algicola*, type specimen. Syntype USNM 35963, ovigerous female, 2.4 mm. (A) Palp of maxilliped. (B) Cheliped, dorsal view. (C) Dactylus and fixed finger of cheliped, ventral view. (D) Pereopod 1. (E) Pereopod 2. (F) Pereopod 3. (G) Pereopod 4. Drawing credit: Jani Jarquín-González.

**Figure 3:**
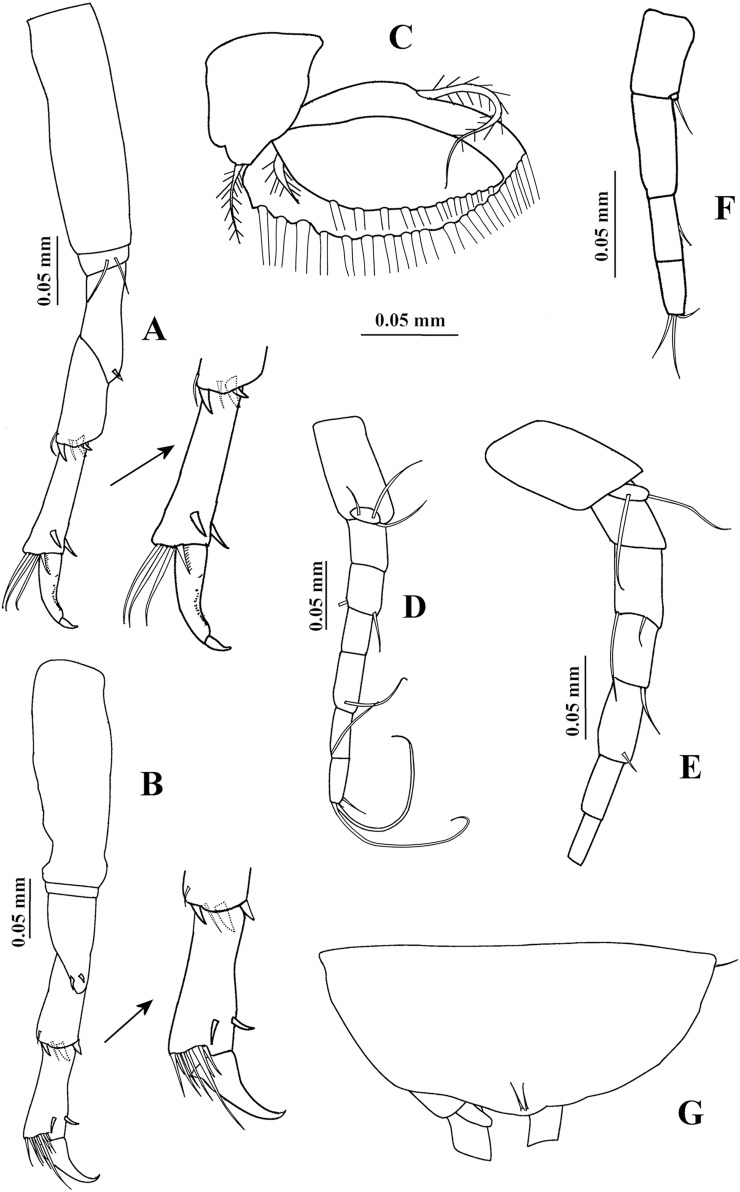
*Chondrochelia algicola*, type and topotype specimens. Syntype USNM 35963, ovigerous female, 2.4 mm. (A) Pereopod 5. (B) Pereopod 6. (C) Pleopod 1. (D) Uropod. (G) Pleotelson. Topotype USNM 35056, non-ovigerous female, 2.2 mm. (E) Uropod. Topotype USNM 35964, juvenile female, 1.5 mm. (F) Uropod. Drawing credit: Jani Jarquín-González.

**Figure 4:**
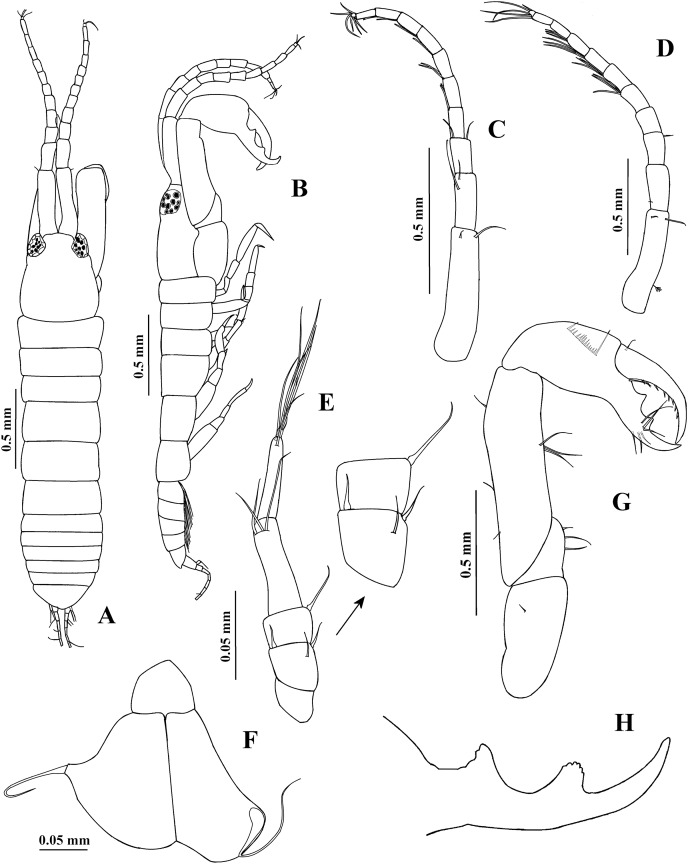
*Chondrochelia algicola*, type and topotype specimens. Syntype USNM 35963, male, 2.3 mm. (A) Habitus, dorsal view. (B) Habitus, lateral view. (D) Antennule. (E) Antenna. (F) Maxilliped. (G) Cheliped. (H) Fixed finger of cheliped. Topotype USNM 35056, male, 2.1 mm. (C) Antennule. Drawing credit: Jani Jarquín-González.

**Figure 5:**
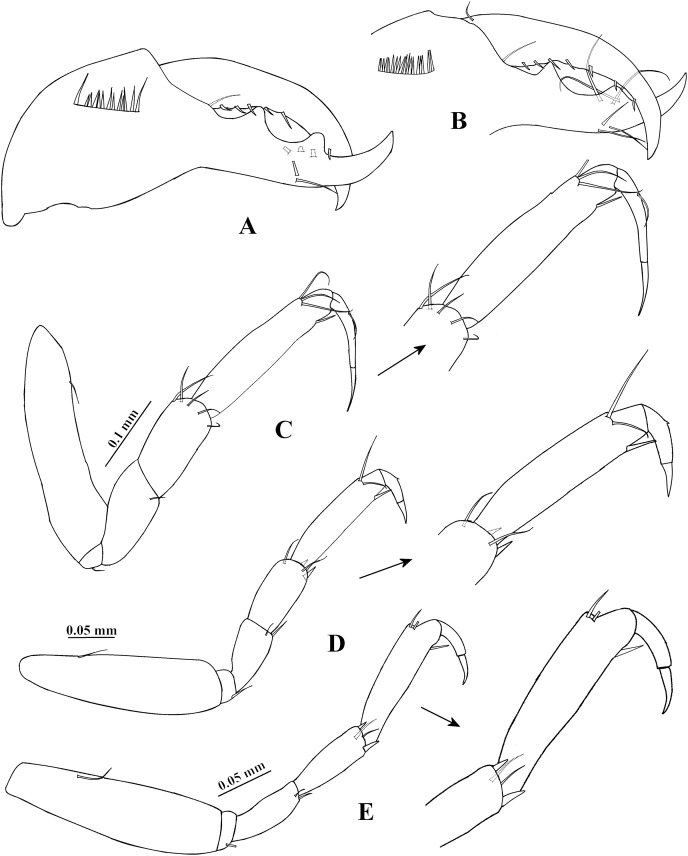
*Chondrochelia algicola*, type and topotype specimens. Topotype USNM 35964, male, 1.9 mm. (A) Dactylus and fixed finger of cheliped, ventral view. Topotype USNM 35056, male, 2.1 mm. (B) Dactylus and fixed finger of cheliped, ventral view. Syntype USNM 35963, male, 2.3 mm. (C) Pereopod 1. (D) Pereopod 2. (E) Pereopod 3. Drawing credit: Jani Jarquín-González.

**Figure 6:**
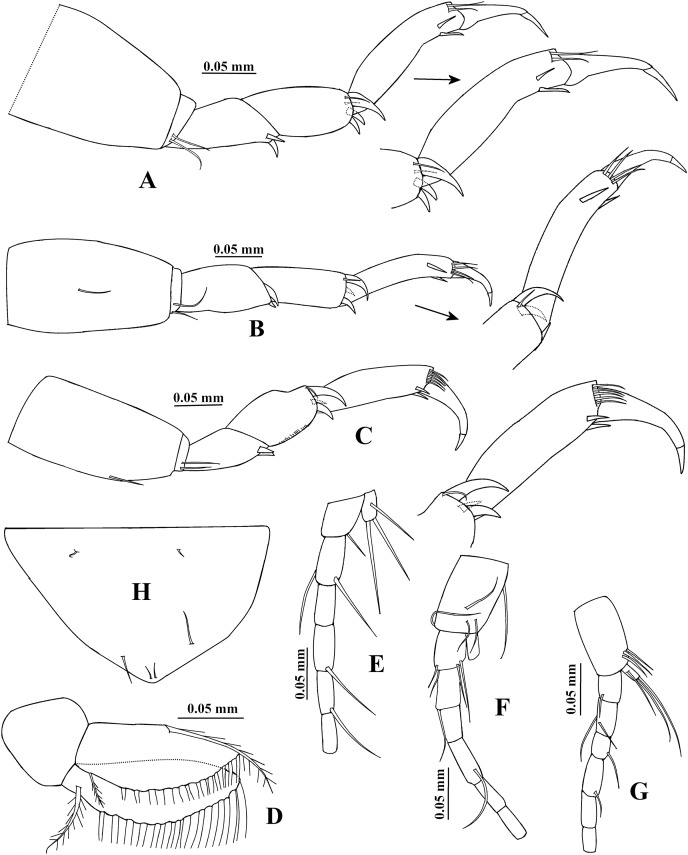
*Chondrochelia algicola*, type and topotype specimens. Syntype USNM 35963, male, 2.3 mm. (A) Pereopod 4. (B) Pereopod 5. (C) Pereopod 6. (D) Pleopod 1. (E) Uropod. (H) Pleotelson. Topotype USNM 35056, male, 2.1 mm. (F) Uropod. Topotype USNM 35964, male, 1.9 mm. (G) Uropod. Drawing credit: Jani Jarquín-González.

*Paratanais algicola Harger, 1878*: 377–378.

*Leptochelia algicola*: Harger, 1879: 162–163, 165; Harger, 1880: 421–424, Figs. 80, 83–86; Anderson, 2020: 445.

*Chondrochelia algicola*: Guţu, 2016: 50, 56.

**Type material**. Syntypes USNM 35963, eight ovigerous females, 27 non-ovigerous females, three males, Long Island Sound, Noank Harbor, Connecticut, USA, in eelgrass, coll. United States Fish Commission, August 1874.

**Additional material**. USNM 35964, 10 ovigerous females, 31 non-ovigerous females, 11 males, eight mancae, same data as syntypes. USNM 35056, four ovigerous females, 19 non-ovigerous females, two males, Buzzards Bay, Naushon Island, Massachusetts, USA, coll. United States Fish Commission, August 20 1887 to August 23 1887.

**Diagnosis**. **Female**. *Mouthparts*. Left mandible with five-denticled *pars incisiva* and smooth *lacinia mobilis*; each maxilliped basis with four setae. *Chelipeds*. Fixed finger cutting edge with five processes; dactylus with smooth cutting edge. *Pereopods*. Pereopod 1 carpus with four distal setae. Pereopod 2 carpus with two setae and one small spine on distal extremity. Pereopod 4 propodus with three dorso-subdistal setae. Pereopod 6 propodus with seven dorso-subdistal setae. **Male**. *Carapace* subrectangular. *Antennules*. Flagellum up to 1.7 times longer than peduncle article-1; with eight articles. *Chelipeds*. Fixed finger cutting edge with two processes separated by a pronounced curvature, proximal longer and pointed, distal apically crenulate. *Pereopods*. Pereopod 1 carpus with five distal setae. Pereopod 2 merus with one slender spine and one seta ventrally. Pereopod 4 propodus with two dorso-subdistal setae. Pereopod 6 propodus with five dorso-subdistal setae. **Both sexes**. *Uropods* with exopod uniarticulate and endopod with six articles.

**Redescription**. **Ovigerous female**. Syntype USNM 35963, 2.4 mm (Figs. 1A, 3G). Body 7.1 times longer than broad. Carapace oval, 1.3 times longer than broad, 0.7 times as long as pereonites 1–3 together; ocular lobes well defined, eyes pigmented. Pereon 2.7 longer than carapace and 4.2 times longer than broad; all pereonites respectively, 1.8, 1.4, 1.3, 1.0, 1.2 and 1.5 times broader than long. Pleon 0.5 times as long as pereonites 1–3 together. Pleotelson 0.3 times as long as pleon; posterior apex with two dorsodistal setules.

Antennule (Fig. 1C) with three long peduncular and one short flagellar article. Article-1 3.5 times longer than broad, with one mesial seta and one distal seta. Article-2 0.3 times as long as article-1, 1.4 times longer than broad, with one distal seta and one sensory seta. Article-3 0.4 times as long as article-1, 3.1 times longer than broad, with two distal setae and one aesthetasc. Article-4 (=flagellum) small, with three setae.

Antenna (Fig. 1D) with six articles. Article-1 0.8 times as long as broad, with one small distal seta. Article-2 1.1 times longer than broad, with one subdistal seta and one slender distal spine on distal extremity, and with slender ventrodistal spine. Article-3 as long as broad, with slender dorsodistal spine. Article-4 3.8 times longer than broad, with one mesial seta and three distal setae. Article-5 0.4 times as long as article-4, 2.5 times longer than broad, with distal seta. Article-6 small, with seven setae.

Labium and labrum (Figs. 1E and 1F) setose, as figured.

Mandibles. *Pars molaris* well developed in both mandibles, with strong rugosity on masticatory surface. Left mandible (Fig. 1G) with five denticled *pars incisiva*; *lacinia mobilis* stout and smooth. *Pars incisiva* of right mandible (Fig. 1H) bifid and distally crenulate.

Maxillule (Fig 1I) with ten robust distal spines and numerous setae on distal extremity and laterally; palp with two distal setae (not illustrated).

Maxilliped (Figs. 1J, 2A). Basis with four long setae on distal extremity extending to palp article-2. Palp article-1, about as long as endite, naked; article-2 with one dorsal seta and four ventro-subdistal setae; article-3 largest, with eight setae ventrally; article-4 with three mesial setae and six distal setae. Endites with two setae, scales (= microtrichia), and three flat spines (two long, pointed, and one short, relatively round) on distal extremity.

Cheliped (Figs. 2B and 2C) basis 1.6 times longer than broad, with one dorso-subdistal simple seta. Merus with three ventral setae. Carpus, 1.7 times longer than broad, with two dorsoproximal small spines, one dorsodistal short seta and three ventrodistal setae. Propodus with one dorsal seta near dactylus articulation, comb-row with five ventral spines and scales. Fixed finger cutting edge with five processes; with six setae, three ventral. Dactylus with ventroproximal seta, and smooth cutting edge.

Pereopod 1 (Fig. 2D) basis 3.5 times longer than broad. Ischium with ventral seta. Merus with oblique articulation with carpus, 2.4 times longer than broad, with ventrodistal seta. Carpus 0.7 times as long as merus, 1.8 times longer than broad, with four distal setae. Propodus 0.7 times as long as merus and carpus together, 3.7 times longer than broad, with three dorso-subdistal and one ventro-subdistal setae. Dactylus as long as carpus, 1.8 times longer than unguis, and with dorsoproximal seta; together with unguis as long as propodus.

Pereopod 2 (Fig. 2E) smaller than pereopod 1, basis 2.8 times longer than broad, with dorsoproximal seta. Ischium with ventral seta. Merus short, 1.7 times longer than broad, with ventrodistal slender spine and ventrodistal seta. Carpus as long as merus, with two setae and one small spine on distal extremity. Propodus 0.6 times as long as basis, with two dorso-subdistal setae and one ventro-subdistal slender spine. Dactylus naked, 1.3 times longer than unguis, together with unguis 0.5 times as long as propodus.

Pereopod 3 (Fig. 2F) similar to pereopod 2, but propodus with three dorso-subdistal setae and one ventro-subdistal spine.

Pereopod 4 (Fig. 2G) basis 3.8 times longer than broad. Ischium with two ventral setae. Merus with oblique articulation with carpus, 2.5 times longer than broad, with two small ventrodistal spines. Carpus as long as merus, with two setae and three spines on distal extremity. Propodus 0.9 times as long as carpus, but narrower, with three dorso-subdistal setae and two ventro-subdistal spines. Dactylus naked, 3.2 times longer than unguis, together with unguis 0.7 times as long as propodus.

Pereopod 5 (Fig. 3A) similar to pereopod 4, but propodus with three setae and one pappose seta on distal extremity.

Pereopod 6 (Fig. 3B) similar to pereopods 4 and 5, but propodus with seven dorso-subdistal setae and two ventro-subdistal spines. Dactylus and unguis together about 0.6 times as long as propodus.

Pleopod 1 (Fig. 3C). Peduncle with one ventrodistal circumplumose seta. Endopod with one middorsal circumplumose seta, one proximoventral circumplumose seta and 11 ventral plumose setae. Exopod with one proximoventral circumplumose seta and 21 ventral plumose setae.

Uropod (Fig. 3D). Exopod uniarticulate, 0.5 times as long as endopod article-1, with one lateral and two terminal setae. Endopod with six articles; articles 1 and 2 broader than other articles, article-2 with two distal setae; article-3 shorter than article-4; article-4 longer than other articles, with subdistal seta; articles 5 and 6 thinner than other articles, with one subdistal seta and three distal setae, respectively.

**Adult male**. Syntype USNM 35963, 2.3 mm (Figs. 4A and 4B, 6H). Body about five times longer than broad. Carapace subrectangular, 1.2 times longer than broad, as long as the pereonites 1–3 together and 1.2 times longer than pleon; ocular lobes well defined, eyes pigmented. Pereon 2.2 times longer than carapace and 2.5 times longer than broad; all pereonites respectively, 3.4, 2.8, 3.4, 2.0, 2.0 and 2.3 times broader than long. Pleon slightly shorter than pereonites 1–3 together. Pleotelson 0.3 times as long as pleon, with four dorsolateral setae; posterior apex similar to female.

Antennule (Fig. 4D) 0.6 times as long as body. Article-1 0.7 times as long as carapace and 4.4 times longer than broad, with two distal setae and one subproximal sensory seta. Article-2 0.4 times as long as article-1, 2.1 times longer than broad, with one proximal seta. Article-3 0.3 times as long as article-1, 1.7 times longer than broad, with one distal seta. Flagellum 1.7 times longer than peduncle article-1, with eight articles; each flagellar articles with three or four aesthetascs; last article minute, with three distal setae.

Antenna (Fig. 4E) with article-1 1.3 times longer than broad, naked. Article-2 1.1 times longer than broad, with two distal spines and one seta. Article-3 0.7 times as long as broad, with one dorsodistal spine. Article-4 3.6 times longer than broad, with three dorsodistal setae. Article-5 0.8 times as long as article-4, 4.8 times longer than broad, with two dorsodistal setae. Article-6 small, with four distal setae.

Mouthparts reduced (Fig. 4F). Maxilliped rudimentary; maxillule palp with one seta.

Cheliped (Figs. 4G and 4H) stout, 0.7 times as long as body. Basis twice as long as broad, with one small dorso-subdistal seta. Merus wider distally, with three ventral setae. Carpus about four times longer than broad, with three ventral setae and three dorsal setae. Propodus with dorsal seta near dactylus articulation, comb-row with 18 ventral setae. Fixed finger cutting edge with two processes separated by a pronounced curvature, proximal process longer and pointed, distal process shorter and apically crenulate; with six setae, three ventral. Dactylus cutting edge with one proximoventral seta and eight spinules.

Pereopod 1 (Fig. 5C) basis about five times longer than broad, with one dorsoproximal seta. Ischium with ventral seta. Merus as long as carpus, twice as long as broad, with one ventrodistal seta. Carpus about twice as long as broad, with five distal setae on distal extremity. Propodus 0.9 times as long as merus and carpus together, 4.6 times longer than broad, with three dorso-subdistal setae and one ventro-subdistal slender spine. Dactylus similar to female, but together with unguis 0.7 times as long as propodus.

Pereopod 2 (Fig. 5D) basis 3.5 times longer than broad, with one dorsoproximal seta. Ischium with ventral seta. Merus as long as carpus, 2.2 times longer than broad, with one slender spine and one seta on ventral side. Carpus 2.1 times longer than broad, with two setae and two spines on distal extremity. Propodus 1.7 times longer than carpus, 4.3 times longer than broad, with two dorso-subdistal setae and one ventro-subdistal slender spine. Dactylus naked, 0.4 times as long as carpus, 1.7 times longer than dactylus, together with unguis 0.5 times as long as propodus.

Pereopod 3 (Fig. 5E) similar to pereopod 2, but merus and carpus subequal.

Pereopod 4 (Fig. 6A) with broad basis. Ischium with two ventral setae. Merus as long as carpus, with two ventral spines. Carpus 2.3 times longer than broad, with two setae and three stout spines on distal extremity. Propodus 3.8 times longer than broad, with two dorso-subdistal setae and two ventro-subdistal spines. Dactylus naked, 0.6 times as long as carpus, 1.2 times longer than unguis, together with unguis 0.7 times as long as propodus.

Pereopod 5 (Fig. 6B) similar to pereopod 4, but basis with one middorsal seta. Carpus with one seta and three stout spines on distal extremity. Propodus five times longer than broad, with four dorsodistal setae and two ventro-subdistal spines.

Pereopod 6 (Fig. 6C) similar to pereopods 4 and 5, but propodus with five dorso-subdistal setae and two ventro-subdistal spines.

Pleopod 1 (Fig. 6D). Endopod with one middorsal circumplumose seta, one proximoventral circumplumose seta and 13 ventral plumose setae. Exopod with one proximoventral circumplumose seta and 16 ventral plumose setae.

Uropod (Fig. 6E). Exopod uniarticulate, 0.8 times as long as endopod article-1, with one lateral and two terminal setae. Endopod with six articles; article-1 broader than other articles, with one distal setae; article-2 longer than other articles, with two distal setae; articles 3 and 6 of similar length; articles 4 and 5 with subequal length, each with one distal seta.

**Variability**. Juvenile female, USNM 35964, 1.5 mm (Fig. 1B). Endopod of uropod with three articles (Fig. 3F). In some adult females the uropod endopod articles can be thicker (Fig. 3E). Male, USNM 35056, 2.1 mm, peduncle articles 2 and 3 of the antennule with two distal setae (Fig. 4C); cheliped dactylus cutting edge with seven spinules (Fig. 5B); uropod peduncle with five setae (Fig. 6F). Male, USNM 35964, 1.9 mm. Antennule flagellum with seven articles (not illustrated). Cheliped dactylus cutting edge with six spinules (Fig. 5A); uropod peduncle with three setae (Fig. 6G).

**Distribution**. Northwestern Atlantic (Massachusetts and Connecticut, USA).

**Type locality**. Long Island Sound, Noank Harbor, Connecticut, USA.

**Habitat**. Shallow-water, in eelgrass and algae.

**Remarks**. Comparing *Chondrochelia algicola* with other members of the genus *Chondrochelia*, we observed morphological similarities to *Chondrochelia savignyi* as redescribed by Bamber (2010) from Madeira, Portugal. In males of both species, the body is about five times longer than broad, the carapace is subrectangular, the chelipeds are small and slender, the pereopod 1 propodus has four distal setae, the uropod exopod is uniarticulate, and the endopod has six articles. However, they differ because *C. algicola* has an antennule 0.6 times as long as the body, while in *C. savignyi* it is shorter, 0.4 times as long; in *C. algicola* the chelipeds are 0.7 times as long as body, but 0.5 times in *C. savignyi*; *C. algicola* has five distal setae on the pereopod 1 carpus, while *C. savignyi* has only two; *C. algicola* has one ventro-subdistal slender spine on the pereopod 2 propodus, but it is absent in *C. savignyi*; *C. algicola* has two dorsodistal setae on the propodus of pereopod 4, whereas *C. savignyi* has four setae. Regarding females, *C. algicola* has an oval carapace, while in *C. savignyi* it is subrectangular; in *C. algicola* the spine of antenna article-3 is 0.5 times as long as anntenal article-4, whereas in *C. savignyi* such spine is 0.3 times as long; *C. algicola* has the left mandible with a stout and smooth *lacinia mobilis*, while in *C. savignyi* it is distally crenulate; *C. algicola* has two distal setae and three flat spines on the maxillipedal endite, whereas *C. savignyi* has a single distal seta and three spatulate spines; *C. algicola* has two setae and three distal spines on the pereopod 4 carpus, while *C. savignyi* has only one seta and four spines; lastly, *C. algicola* has a pappose seta on the pereopod 5 propodus, which is lacking in *C. savignyi*.

***Chondrochelia caribensis* sp. nov.**

urn:lsid:zoobank.org:act:45FD18D0-1D0F-4921-98DD-3154A13460A7

Figures 7–13

**Figure 7:**
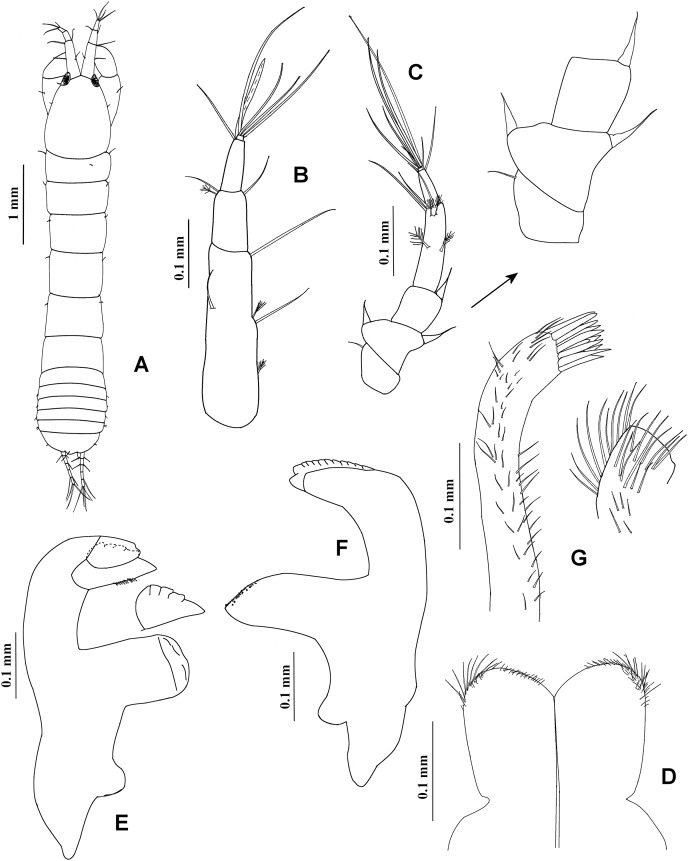
*Chondrochelia caribensis* sp. nov. Holotype ECOSUR 235, non-ovigerous female, 4.6 mm. (A) Habitus. (B) Antennula. (C) Antenna. (D) Labium. (E) Left mandible. (F) Right mandible. (G) Maxillule. Drawing credit: Jani Jarquín-González.

**Figure 8:**
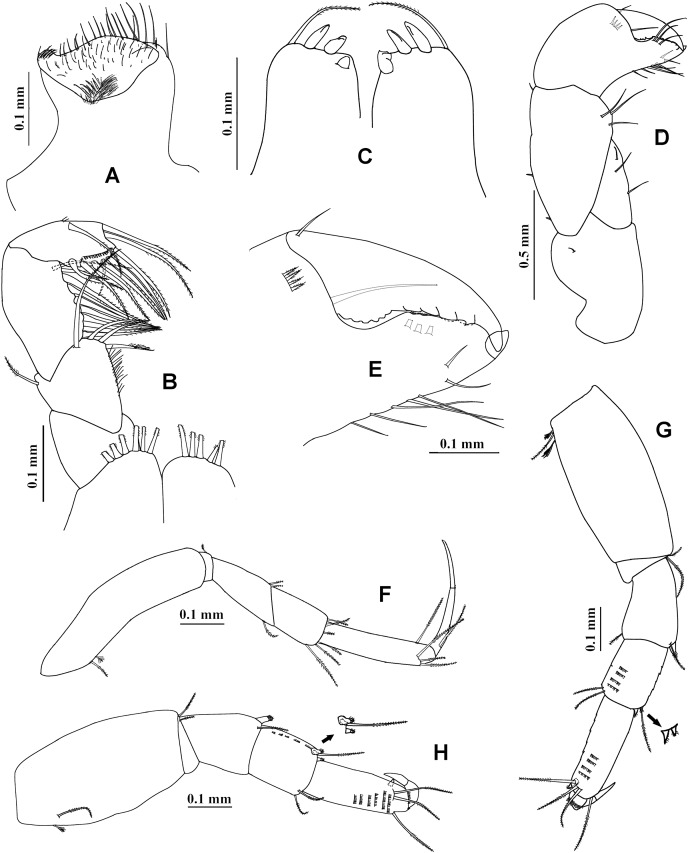
*Chondrochelia caribensis* sp. nov. Holotype ECOSUR 235, non-ovigerous female, 4.6 mm. (A) Labrum. (B) Basis and palp of maxilliped. (C) Endite of maxilliped. (D) Cheliped. (E) Dactylus and fixed finger of cheliped, ventral view. (F) Pereopod 1. (G) Pereopod 2. (H) Pereopod 3. Drawing credit: Jani Jarquín-González.

**Figure 9:**
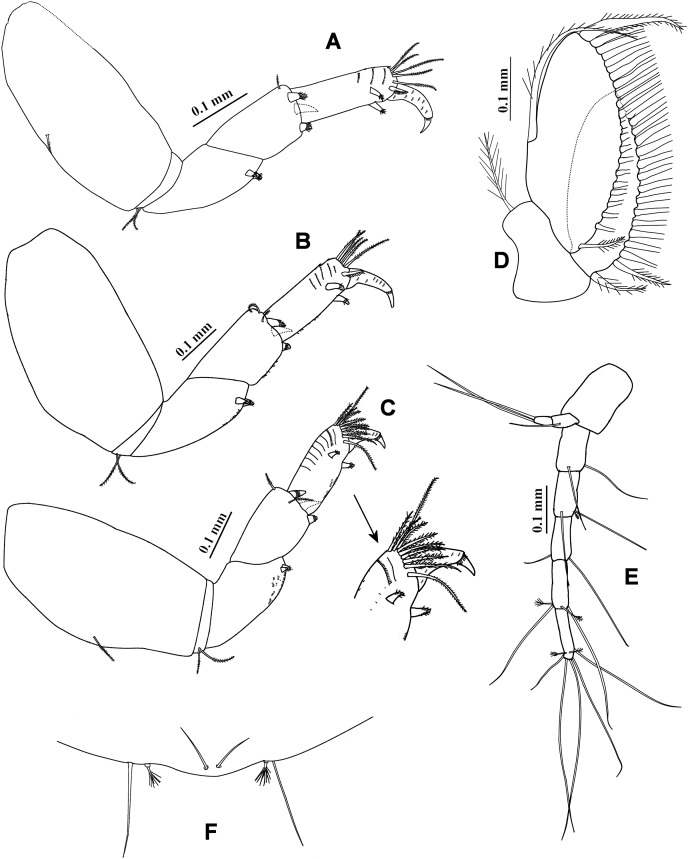
*Chondrochelia caribensis* sp. nov. Holotype ECOSUR 235, non-ovigerous female, 4.6 mm. (A) Pereopod 4. (B) Pereopod 5. (C) Pereopod 6. (D) Pleopod 1. (E) Uropod. (F) Posterior apex of pleotelson. Drawing credit: Jani Jarquín-González.

**Figure 10:**
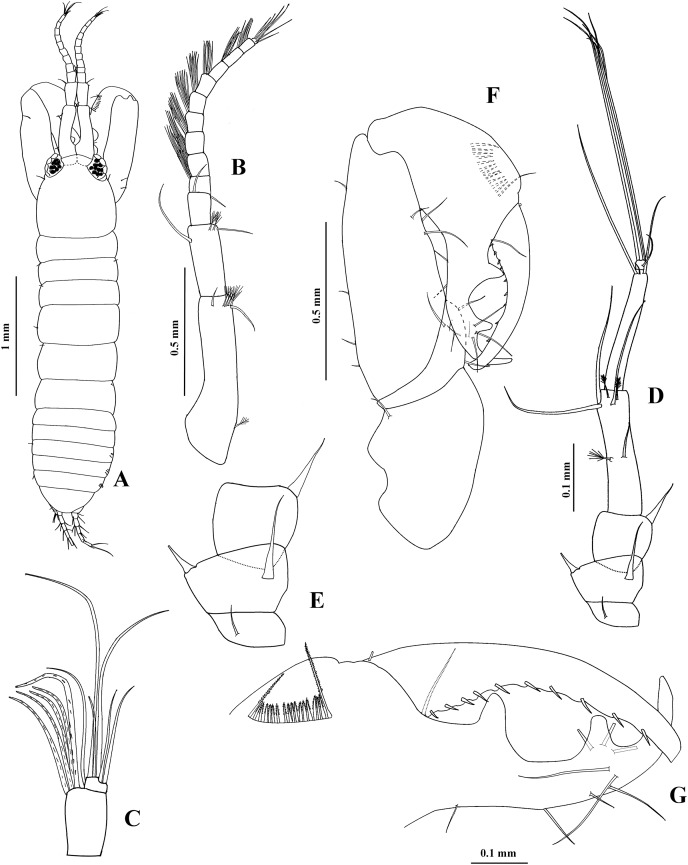
*Chondrochelia caribensis* sp. nov. Paratype ECOSUR 236, male, 3.0 mm. (A) Habitus. (B) Antennula. (C) Last two articles of antennule. (D) Antenna. (E) First three articles of antenna. (F) Cheliped. (G) Dactylus and fixed finger of cheliped, ventral view. Drawing credit: Jani Jarquín-González.

**Figure 11:**
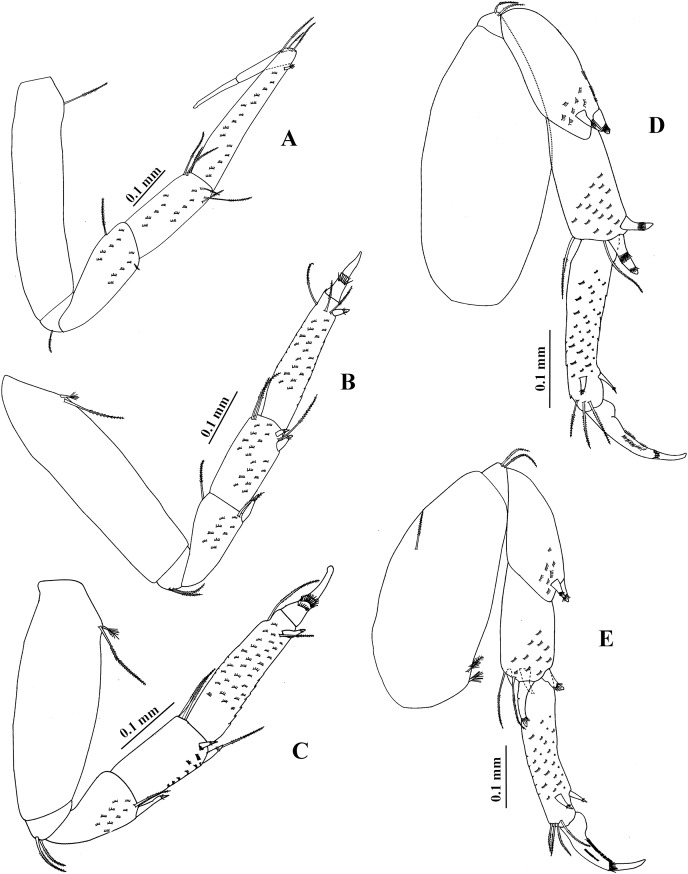
*Chondrochelia caribensis* sp. nov. Paratype ECOSUR 236, male, 3.0 mm. (A) Pereopod 1. (B) Pereopod 2. (C) Pereopod 3. (D) Pereopod 4. (E) Pereopod 5. Drawing credit: Jani Jarquín-González.

**Figure 12:**
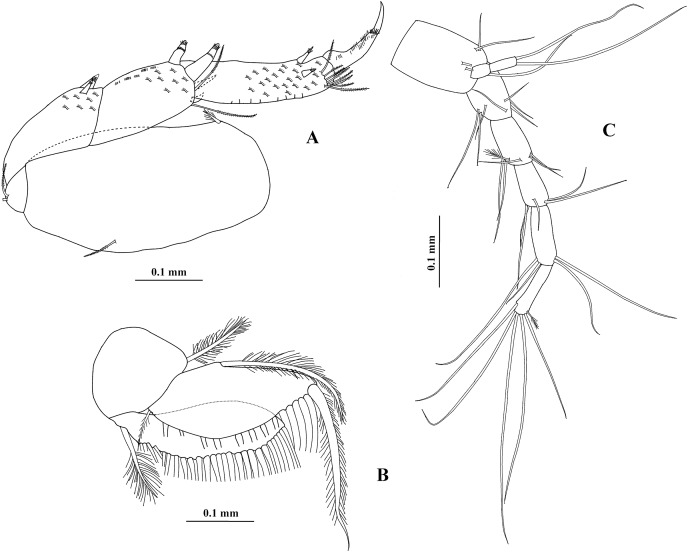
*Chondrochelia caribensis* sp. nov. Paratype ECOSUR 236, male, 3.0 mm. (A) Pereopod 6. (B) Pleopod 1. (C) Uropod. Drawing credit: Jani Jarquín-González.

**Figure 13:**
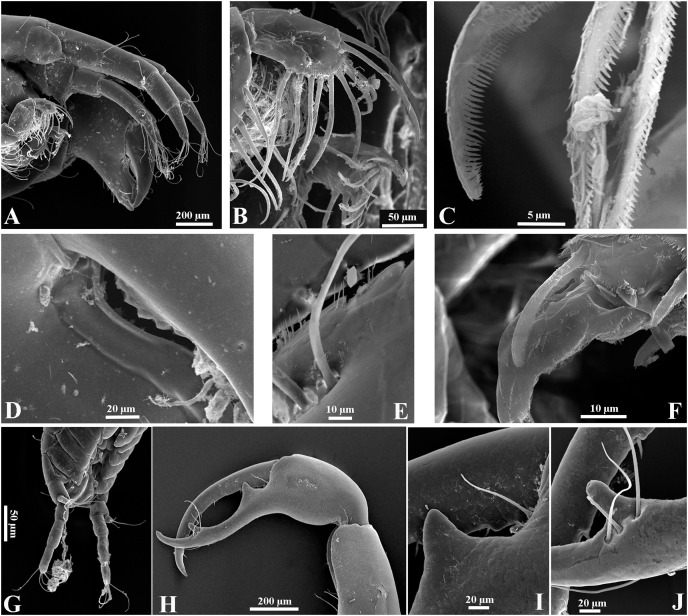
*Chondrochelia caribensis* sp. nov. Topotype specimens. Females, male and manca SEM images. Non-ovigerous female, 3.8 mm, ECOSUR. (A) Antennule and antenna. (B) Plumose setae on fourt palp of maxilliped. (C) Plumose setae on propodus of pereopod 4. Ovigerous female, 3.5 mm, ECOSUR. (D) Dactylus of cheliped with crenulate cutting edge. (E) Fixed finger of cheliped with setulate processes on cutting edge. Manca, 0.6 mm, ECOSUR. (F) Plumose setae and scales on propodus of pereopod 4. (G) Uropods. Male, 2.5 mm, ECOSUR. (H) Dactylus and propodus of cheliped. (I) Proximal process on cutting edge of fixed finger of cheliped. (J) Distal process on cutting edge of fixed finger of cheliped. Photo credit: Luis Fernando Carrera-Parra.

*Leptochelia dubia*: Suárez-Morales et al., 2004: 54–60, figs. 25–28; García-Madrigal, Heard & Suárez-Morales, 2005: 1158–1160, 1166.

**Type material**. Holotype ECOSUR 235, non-ovigerous female, 4.6 mm, Mahahual, Quintana Roo, Mexico, 18°43′20.47″N 87°42′5.43″W, 1.3 m, in *Thalassia testudinum* and *Syringodium filiforme*, June 5 1998. Paratypes: ECOSUR 236, one ovigerous female, one adult male, same data as holotype. ECOSUR 237, one ovigerous female, 30 non-ovigerous females, one male, four mancae, Xahuayxol, Quintana Roo, Mexico, 18°30′12.00″N 87°45′28″W, 1 m, in calcareous algae, May 16 2015.

**Additional material**. ECOSUR-C1081, one ovigerous female, five non-ovigerous females, one manca, Xcacel, Quintana Roo, Mexico, 20°20′27.6″N 87°20′24″W, 1.5 m, in algae, June 25 2014. ECOSUR-C1082, 10 non-ovigerous females, Mahahual, Quintana Roo, Mexico, 18°43′20.85″N 87°42′3.84″W, in coralline rock, May 15 1999. ECOSUR-C1083, two non-ovigerous females, three adult males, Rancho Buenavista, Xahuayxol, Quintana Roo, Mexico, 18°30′42.00″N 87°45′27.17″W, in *Thalassia testudinum*, June 06 1998. ECOSUR-C1084, 10 ovigerous females, 31 non-ovigerous females, seven mancae, Xahuayxol, Quintana Roo, Mexico, 18°29′13.2″N 87°45′25.2″W, 1 m, in algae, May 2 2013. ECOSUR-C1085, three non-ovigerous females, one adult male, five mancae, Xahuayxol, Quintana Roo, Mexico, 18°30′12.00″N 87°45′28″W, 1 m, in calcareous algae, August 07 2015.

**Molecular material**. ECOSUR TANAI040-15, TANAI041-15 (one non-ovigerous female, one non-ovigerous female): Xcacel, 20°20′27.6″N 87°20′24″W, Quintana Roo, Mexico, 1.5 m, in algae, June 25 2014.

**Diagnosis**. **Female**. *Mouthparts*. Left mandible with weakly bifid *pars incisiva*, *lacinia mobilis* with four outer denticles and inner setules; maxilliped left basis with six plumose setae, right basis with five plumose setae. *Chelipeds*. Fixed finger cutting edge with three setulate processes; dactylus cutting edge crenulate proximally and with four distal processes. *Pereopods*. Pereopod 1 carpus with six distal plumose setae. Pereopod 2 carpus with three setae and two spines on distal extremity. Pereopod 4 propodus with five plumose setae and one pappose seta on distal extremity. Pereopod 6 propodus with 10 dorso-subdistal plumose setae. **Male**. *Carapace* round. *Antennules*. Flagellum as long as peduncle article-1; with 10 articles. *Chelipeds*. Fixed finger cutting edge with two processes, proximal broad and pointed, distal narrow and blunt. *Pereopods*. Pereopod 1 carpus with six distal plumose setae. Pereopod 2 merus with one dorso-subdistal plumose seta, with one setulate spine and one plumose seta on ventral side. Pereopod 4 propodus with three dorso-subdistal plumose setae. Pereopod 6 propodus with eight dorso-subdistal plumose setae. **Both sexes**. *Uropods* with exopod biarticulate and endopod with five articles.

**Etymology**. The name of the species refers to the geographic region where specimens were collected.

**Description**. **Non-ovigerous female**. Holotype ECOSUR 235, 4.6 mm (Figs. 7A, 9F). Body seven times longer than broad. Carapace oval, 1.3 times longer than broad, 0.9 times as long as pereonites 1–3 together. Ocular lobes well defined, eyes pigmented. Pereon 2.4 times longer than carapace and 3.9 times longer than broad; all pereonites respectively, 2.7, 2.0, 1.5, 1.2, 1.5 and 2.0 times broader than long. Pleon 0.7 times as long as pereonites 1–3 together. Pleotelson 0.3 times as long as pleon, posterior apex with four simple setae and two sensory setae.

Antennule (Fig. 7B) with three long peduncular and one short flagellar articles. Article-1 3.7 times longer than broad, with two mesial setae, one distal simple seta, and two sensory setae (one subproximal and other mesial). Article-2 0.3 times as long as article-1, 1.8 times longer than broad, with two simple setae and one sensory distal seta. Article-3 0.3 times as long as article-1, 2.7 times longer than broad, with two distal simple setae and one aesthetasc. Article (= flagellum) small, with three distal simple setae.

Antenna (Fig. 7C) with six articles. Article-1 longer than broad, with one distal simple seta. Article-2 0.8 times as long as broad, narrow distally, with two stout distal spines. Article-3 about as long as broad, with one stout distal spine. Article-4 3.6 times longer than broad, with two mesial, two distal sensory setae, and three simple setae distally. Article-5 0.4 times as long as article-4, 2.6 times longer than broad, with two simple distal setae. Article-6 small, with six simple setae.

Labium and labrum (Figs. 7D, 8A) setose, as figured.

Mandibles. *Pars molaris* well developed in both mandibles, with rugosity on masticatory surface. Left mandible (Fig. 7E) with *pars incisiva* stout and weakly bifid; *lacinia mobilis* with four outer denticles and setules on inner margin. *Pars incisiva* of right mandible (Fig. 7F) bifid and distally crenulate.

Maxillule (Fig. 7G) with 11 robust spines distally and numerous setae laterally and on distal extremity.

Maxilliped (Figs. 8B and 8C). Left basis with six plumose setae, and right basis with five plumose setae on distal extremity. Palp article-1, about as long as endite, naked; article-2 with one dorsal plumose seta and four ventrodistal plumose setae, mesial margin finely setulate; article-3 largest, with 10 plumose setae ventrally; article-4 with one mesial plumose seta, seven ventrodistal plumose setae, two plumose setae distally, and scales. Endites with one plumose seta and three flat spines (two longer and one short, relatively round) on distal extremity.

Cheliped (Figs. 8D and 8E) basis 1.4 times longer than broad, with one small dorso-subdistal simple seta. Merus with three simple ventral setae. Carpus, 1.8 times longer than broad, with two small dorsoproximal spines, one short dorsodistal seta and three ventrodistal setae. Propodus with one dorsal simple seta near dactylus articulation, comb-row with four ventral setulate spines and scales. Fixed finger cutting edge with three setulate processes; with 11 simple setae, six ventral. Dactylus with ventroproximal simple seta, cutting edge crenulate proximally and with four broad distal processes.

Pereopod 1 (Fig. 8F) basis 4.3 times longer than broad, with one plumose seta and one sensory seta subproximally. Ischium with ventral plumose seta. Merus with oblique articulation with carpus, 2.7 times longer than broad, with one dorso-subdistal plumose seta and two ventrodistal plumose setae. Carpus 1.6 times longer than broad, with six plumose setae on distal extremity. Propodus 0.7 times as long as merus and carpus together, 4.8 times longer than broad, with three dorso-subdistal plumose setae and one ventro-subdistal plumose seta. Dactylus about as long as carpus, with dorsoproximal plumose seta; together with unguis as long as propodus.

Pereopod 2 (Fig. 8G) basis twice as long as broad, with one proximal plumose seta and two proximal sensory setae. Ischium with two ventral plumose setae. Merus 1.8 times longer than broad, with two plumose setae and one setulate spine on distal extremity. Carpus 0.7 times as long as merus, with three plumose setae, and two small setulate spines and scales on distal extremity. Propodus about 0.6 times as long as basis, with four distal plumose setae, one subdistal setulate spine and scales. Dactylus with scales, 1.1 times longer than unguis, together with unguis approximately 0.5 times as long as propodus.

Pereopod 3 (Fig. 8H) similar to pereopod 2 but smaller, with one proximal plumose seta and one sensory seta. Merus without dorsodistal plumose seta. Propodus with three distal plumose setae, one subdistal setulate spine and scales.

Pereopod 4 (Fig. 9A) basis twice as long as broad, with one subventral plumose seta. Ischium with two ventral plumose setae. Merus with oblique articulation with carpus, 2.2 times longer than broad, with two ventro-subdistal setulose spines. Carpus 0.7 times as long as merus, with one plumose seta and three setulate spines on distal extremity. Propodus as long as carpus, with five distal plumose setae, one distal pappose seta, two ventro-subdistal setulate spines, and scales. Dactylus with scales, 2.8 times longer than unguis, together with unguis 0.6 times as long as propodus.

Pereopod 5 (Fig. 9B) similar to pereopod 4, but carpus with two plumose setae and three setulate spines on distal extremity.

Pereopod 6 (Fig. 9C) similar to pereopods 4 and 5, but propodus with 10 dorso-subdistal plumose setae and two ventro-subdistal setulate spines.

Pleopod 1 (Fig. 9D). Peduncle with one dorso-subdistal circumplumose seta. Endopod with one middorsal circumplumose seta, one proximoventral circumplumose seta, and 19 ventral plumose setae. Exopod with proximoventral circumplumose seta and 29 ventral plumose setae.

Uropod (Fig. 9E). Exopod 0.8 times as long as endopod article-1; biarticulate, article-1 small and with one distal simple seta with terminal pore, article-2 with two apical simple setae with terminal pore. Endopod with five articles; articles 1 and 2 broader than other articles; articles 1 and 3 with two simple setae with terminal pore; article-2 with two simple setae with terminal pore and one sensory seta; article-4 with two sensory setae and two simple setae with terminal pore; article-5 with two sensory setae and five simple setae with terminal pore.

**Adult male**. Paratype ECOSUR 236, 3.0 mm (Fig. 10A). Body 4.3 times longer than broad. Carapace round, about as long as broad, 1.2 times longer than pereonites 1–3 together and about as long as pleon. Ocular lobes well defined, eyes pigmented. Pereon 2.3 times longer than carapace and 2.3 times longer than broad; all pereonites respectively, 4.2, 4.0, 3.4, 2.1, 2.3 and 2.8 times broader than long. Pleon almost as long as pereonites 1–3 together. Pleotelson 0.4 times as long as pleon, similar to female.

Antennule (Figs. 10B and 10C) 0.4 times as long as body. Article-1 0.7 times as long as carapace, five times longer than broad, with one subproximal sensory seta, two simple setae and four sensory setae on subdistal extremity. Article-2 0.4 times as long as article-1, 2.5 times longer than broad, with two simple setae and two sensory setae on subdistal extremity. Article-3 0.2 times as long as article-1, 1.7 times longer than broad, with two simple distal setae. Flagellum as long as peduncle article-1, with ten articles; each flagellar article with at least five aesthetascs; last article minute, with six distal simple setae.

Antenna (Figs. 10D and 10E) article-1 twice as broad as long, with one subproximal simple seta. Article-2 about 0.7 times as long as broad, with two stout distal spines. Article-3 as long as broad, with one distal stout spine. Article-4 4.3 times longer than broad, with one mesial sensory seta and one mesial simple seta, three distal simple setae, and two distal sensory setae. Article-5 longer than article-4, 6.2 times longer than broad, with three distal simple setae, one of them small. Article-6 small, with six distal simple setae.

Mouthparts reduced. Maxilliped rudimentary (not illustrated).

Cheliped (Figs. 10F and 10G) stout, 0.7 times as long as body. Basis 1.6 times longer than broad, with one dorsodistal simple seta. Merus wider distally, with three ventral setae. Carpus about three times longer than broad, with three ventral setae and four dorsal setae. Propodus with dorsal seta near dactylus articulation, comb-row with 18 ventral setulate spines. Fixed finger cutting edge with two processes separated by a pronounced curvature, proximal broad with pointed tip, distal narrow with blunt tip, with nine simple setae, three dorsal. Dactylus cutting edge with one proximodorsal simple seta and 10 spinules.

Pereopod 1 (Fig. 11A) basis about five times longer than broad, with one dorsoproximal plumose seta. Ischium with ventral plumose seta. Merus 1.2 times longer than carpus, 2.6 times longer than broad, with one dorso-subdistal plumose seta, one small ventral plumose seta, and scales. Carpus 2.6 times longer than broad, with six plumose setae on distal extremity, with scales. Propodus 0.7 times as long as merus and carpus together, 6.5 times longer than broad, with three dorsodistal plumose setae, one ventro-subdistal setule spine, and scales. Dactylus with scales, 1.3 times longer than unguis; together with unguis 0.7 times as long as propodus.

Pereopod 2 (Fig. 11B) basis 3.7 times longer than broad, with one dorsoproximal plumose seta and one dorsoproximal sensory seta. Ischium with two ventral plumose setae. Merus as long as carpus, 2.1 times longer than broad, with one dorso-subdistal plumose seta, and one setulate spine and one plumose seta on ventral side, with scales. Carpus 2.1 times longer than broad, with three plumose setae and two stout setulate spines on distal extremity, with scales. Propodus four times longer than broad, with three dorso-subdistal plumose setae, one ventro-subdistal spine and scales. Dactylus with scales, 0.3 times as long as carpus, 2.1 times longer than unguis, together with unguis 0.4 times as long as propodus.

Pereopod 3 (Fig. 11C) similar to pereopod 2, but propodus with two dorso-subdistal plumose setae and one ventrodistal setulate spine.

Pereopod 4 (Fig. 11D) with broad basis. Ischium with two ventral plumose setae. Merus 1.2 times longer than carpus, with two ventrodistal setulate spines, with scales. Carpus 2.2 times longer than broad, with two plumose setae and two stout setulate spines on distal extremity, with scales. Propodus 5.1 times longer than broad, with three dorso-subdistal plumose setae and two ventro-subdistal stout setulate spines, with scales. Dactylus with scales, 0.6 times as long as carpus, 2.7 times longer than unguis, together with unguis 0.6 times as long as propodus.

Pereopod 5 (Fig. 11E) similar to pereopod 4, but basis with three proximal sensory setae and one dorso-subdistal plumose seta. Carpus with two plumose setae and four stout setulate spines on distal extremity. Propodus with four dorsodistal plumose setae and two ventro-subdistal setulate spines.

Pereopod 6 (Fig. 12A) similar to pereopods 4 and 5, but propodus with eight dorsodistal plumose setae and two ventro-subdistal setulate spines.

Pleopod 1 (Fig. 12B). Peduncle with one dorsoproximal circumplumose seta. Endopod with one middorsal circumplumose seta, one proximoventral circumplumose seta and 16 ventral plumose setae. Exopod with one proximoventral circumplumose seta and 23 ventral plumose setae.

Uropod (Fig. 12C) similar to uropod of female, but peduncle with four distal simple setae with terminal pore; exopod 0.7 times as long as endopod article-1, and endopod articles with more setae.

Additionally, the SEM analysis allowed us to observe in the detail the ornamentation of antennules and antennae (Fig. 13A), maxillipeds (Fig. 13B), setae on the pereopods (Figs. 13C, 13F), the processes on the cutting edge of the cheliped fixed finger and dactylus (Figs. 13D and 13E, 13H–13J), as well as the shape of uropods in mancae (Fig. 13G).

**Variability**. Ovigerous female, ECOSUR-C1084, 6.2 mm. Maxilliped palp with 13 ventral plumose setae on article-3 and 14 ventral plumose setae on article-4. Cheliped propodus comb-row with seven ventral setulate spines. Pereopod 6 propodus with 12 dorso-subdistal plumose setae. Pleopod 1 endopod with 27 ventral plumose setae and exopod with 41 ventral plumose setae. Male, ECOSUR-C1085, 2.3 mm. Antennule flagellum with seven articles. Cheliped dactylus cutting edge with seven ventral spinules. Pereopod 4 propodus with four dorsodistal plumose setae. Pereopod 6 propodus with five dorsodistal plumose setae. Uropod exopod with one article.

**Distribution**. Mexican Caribbean. Quintana Roo, Mexico, from Xcacel to Xahuayxol.

**Type locality**. Mahahual, Quintana Roo, Mexico.

**Habitat**. Shallow-water, in coralline rocks, seagrass, and algae.

**Remarks**. *Chondrochelia caribensis* sp. nov. resembles *Chondrochelia africana* (Larsen & Froufe, 2013) from Guinea Bissau, West Africa, as the males and females of both species have a pereopod 6 propodus with at least seven setae, two of them longer than the others, and the uropod endopod has five articles, the exopod two articles. Also, adult females of both species have stout spines on antenna articles 2 and 3, the pereopod 1 merus has two ventrodistal setae, the pereopod 1 carpus has six distal setae, and the pereopod 1 propodus has four distal setae. However, they differ because females of *C. caribensis* sp. nov. has fine setae on the inner margin of the *pars incisiva* of the left mandible, while in *C. africana* the inner margin lacks these; *C. caribensis* sp. nov. has up to six distal plumose setae on the maxilliped basis, whereas *C. africana* has only three; in *C. caribensis* sp. nov. the cheliped dactylus cutting edge is crenulate proximally and with four distal processes, while in *C. africana* the cutting edge is smooth. Also, in *C. caribensis* sp. nov. the uropod exopod is up to 0.8 times as long as endopod article-1, whereas in *C. africana* the exopod is more than 0.8 times as long. Regarding males, both species are similar in having eight articles in the flagellum of the antennule, two processes on the fixed finger of dactylus separated by a pronounced curvature, and the merus of pereopod 1 with one dorso-subdistal seta and one small subventral seta. However, they differ because in *C. caribensis* sp. nov. the antennule article-1 is up to five times longer than broad, while in *C. africana* it is more than five times longer than broad; in *C. caribensis* sp. nov. the proximal process is larger than the distal process on fixed finger of cheliped, whereas in *C. africana* the proximal process is smaller; in *C. caribensis* sp. nov. uropod exopod is 0.7 times as long as endopod article-1, while in *C. africana* the exopod is 1.7 times longer.

Females of *C. caribensis* sp. nov. and *C. dubia* differ as in *C. dubia* the antennule peduncle article-3 has a length-width ratio of 4.2, while in *C. caribensis* sp. nov. it is stouter, 2.7; in *C. dubia* the length-width ratio of the antenna articles 4 and 5 are 5.0 and 4.5, compared to 3.6 and 2.6 in *C. caribensis* sp. nov. Also, in *C. dubia* the uropod is almost 0.16 times as long as the body, whereas in *C. caribensis* sp. nov. it is less than 0.16 times; *C. dubia* has an uniarticulated uropod exopod, while in *C. caribensis* sp. nov. it is biarticulate. Other differences are shown in Table 2.

**Table 2:** Main differences between females of *Chondrochelia dubia*, *C. caribensis* sp. nov., and *C. winfieldi* sp. nov.

	*C. dubia* Krøyer (1842)	*C. caribensis* n. sp.	*C. winfieldi* n. sp.
Locality	Salvador Bahia, Brazil	Mahahual, Quintana Roo, Mexico	Isla Verde, Veracruz, Mexico
Length	3.5 mm	4.6 mm	3.0 mm
Antennule *vs* body length	Less than 0.16 times as long as body	0.16 times as long as body	0.15 times as long as body
Antennule—Ratio (length/width) of peduncle articles 1–3	3.7 + 2.1 + 4.2*	3.7 + 1.8 + 2.7	3.0 + 1.4 + 2.5
Antenna—Ratio (length/width) of articles 2–5	2.1 + 1.8 + 5.0 + 4.5*	0.8 + 1.0 + 3.6 + 2.6	1.3 + 1.0 + 3.4 + 2.3
Pleon *vs* carapace length	As long as carapace	0.8 times as long as carapace	0.8 times as long as carapace
Uropod *vs* body length	Almost 0.16 times as long as body	0.09 times as long as body	0.1 times as long as body
Uropod—No. of exopod articles	1	2	2
Uropod—Exopod *vs* endopod article-1 length	0.7 times as long as endopod article-1*	0.8 times as long as endopod article-1	0.9 times as long as endopod article-1
Uropod—Endopod, ratio (length/width) of articles 1–5	1.5 + 1.5 + 1.8 + 1.5 + 4.1 + 4.8*	1.6 + 1.8 + 2.0 + 2.2 + 3.0 + 3.8	1.8 + 1.1 + 1.5 + 1.8 + 3.0 + 3.8

**Note:**

* Measurements obtained from figures 20, 21, and 22 of Krøyer (1842).

***Chondrochelia winfieldi* sp. nov.**

urn:lsid:zoobank.org:act:E8BD8518-865F-49AE-90AC-E2F95CD121AF

Figures 14–19

**Figure 14:**
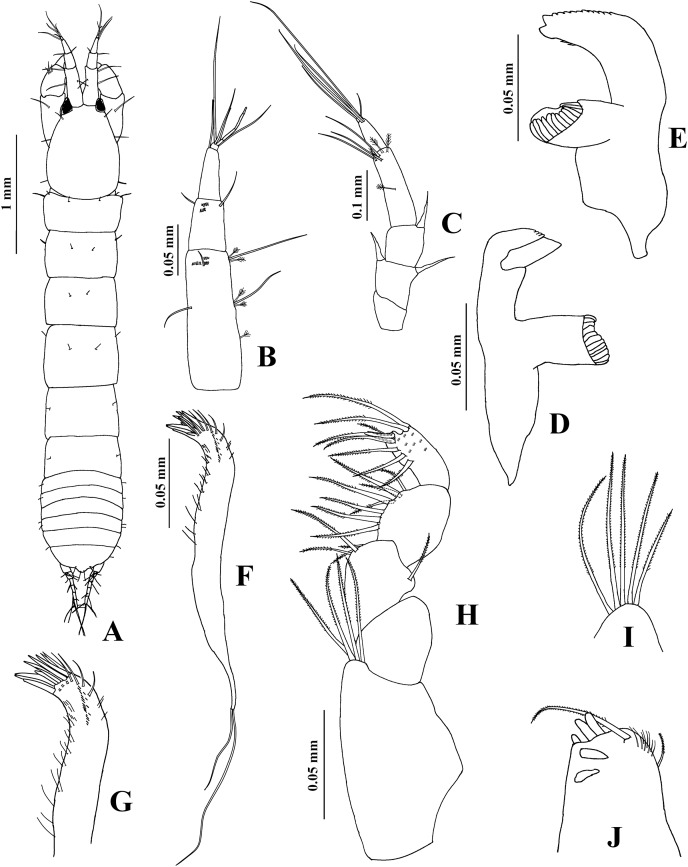
*Chondrochelia winfieldi* sp. nov. Holotype ECOSUR 238, non-ovigerous female, 3.0 mm. (A) Habitus. (B) Antennule. (C) Antenna. (D) Left mandible. (E) Right mandible. (F) Maxillule. (G) Detail of spines and setules of maxillule. (H) Maxilliped. (I) Plumose setae on palp of maxilliped. (J) Endite of maxilliped. Drawing credit: Jani Jarquín-González.

**Figure 15:**
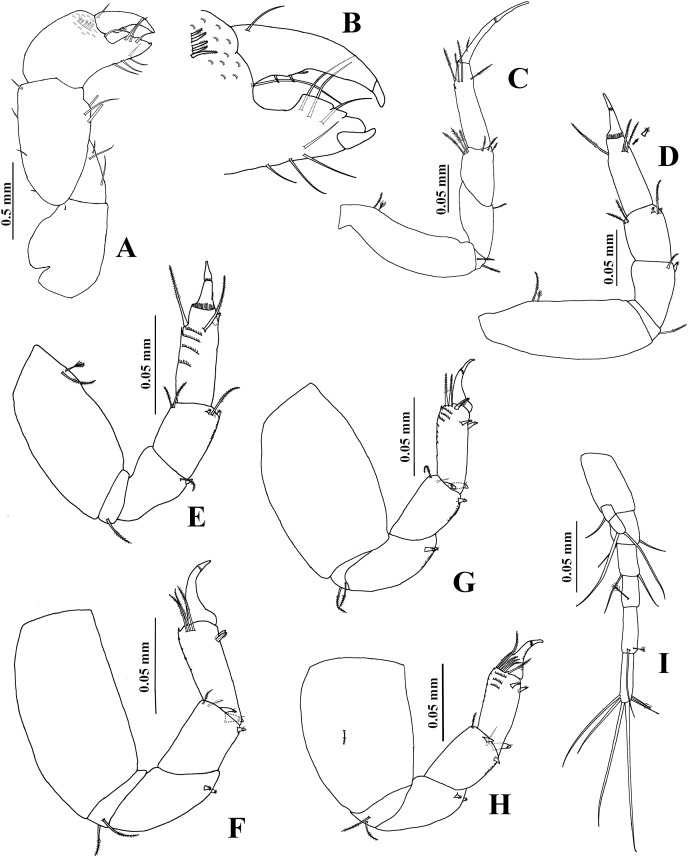
*Chondrochelia winfieldi* sp. nov. Holotype ECOSUR 238, non-ovigerous female, 3.0 mm. (A) Cheliped. (B) Dactylus and fixed finger of cheliped, ventral view. (C) Pereopod 1. (D) Pereopod 2. (E) Pereopod 3. (F) Pereopod 4. (G) Pereopod 5. (H) Pereopod 6. (I) Uropod. Drawing credit: Jani Jarquín-González.

**Figure 16:**
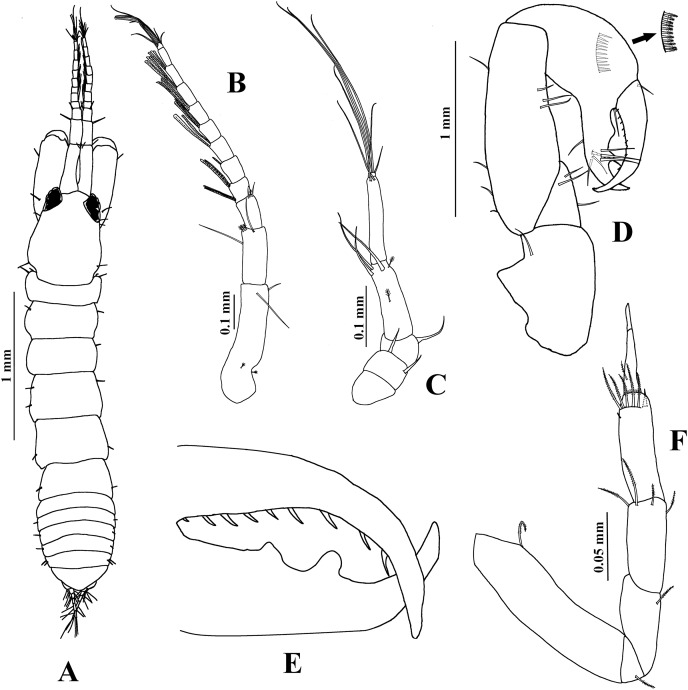
*Chondrochelia winfieldi* sp. nov. Paratype ECOSUR 240, male, 2.6 mm. (A) Habitus. (B) Antennule. (C) Antenna. (D) Cheliped. (E) Dactylus and fixed finger of cheliped, ventral view. (F) Pereopod 1. Drawing credit: Jani Jarquín-González.

**Figure 17:**
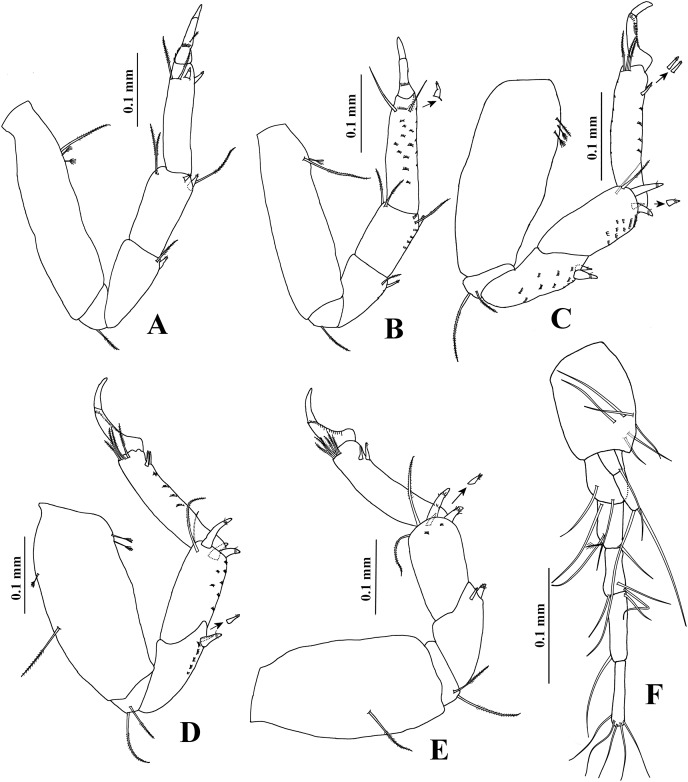
*Chondrochelia winfieldi* sp. nov. Paratype ECOSUR 240, male, 2.6 mm. (A) Pereopod 2. (B) Pereopod 3. (C) Pereopod 4. (D) Pereopod 5. (E) Pereopod 6. (F) Uropod. Drawing credit: Jani Jarquín-González.

**Figure 18:**
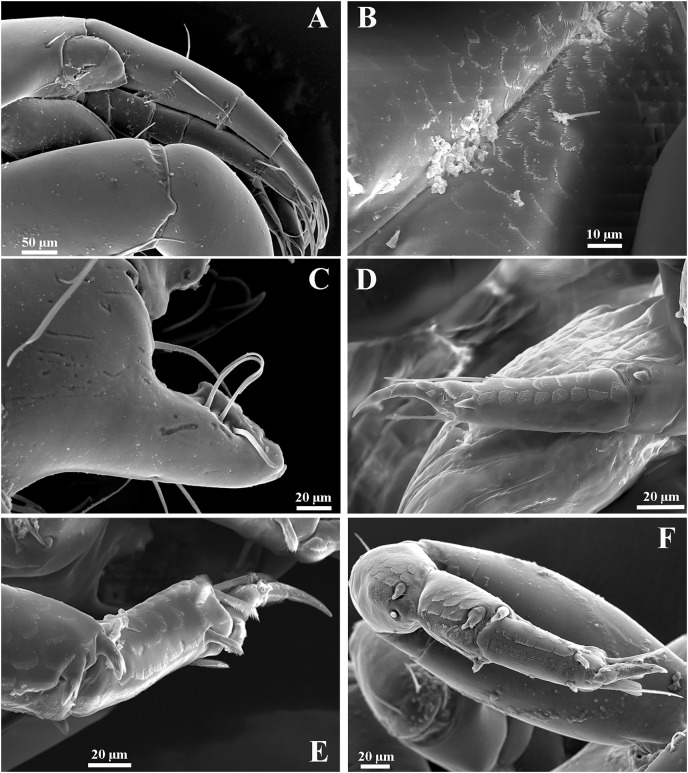
*Chondrochelia winfieldi* sp. nov. Topotype specimens. SEM images. Non-ovigerous female, 2.5 mm, ECOSUR. (A) Antennules. (B) Scales on bases of maxilliped. (C) Detail of cutting edge of cheliped. (D) Scales on propodus of pereopod 3. (E) Detail of the ornamentation of the carpus and propodus of pereopod 5. (F) Detail of the ornamentation of the merus, carpus and propodus of pereopod 6. Photo credit: Luis Fernando Carrera-Parra.

**Figure 19:**
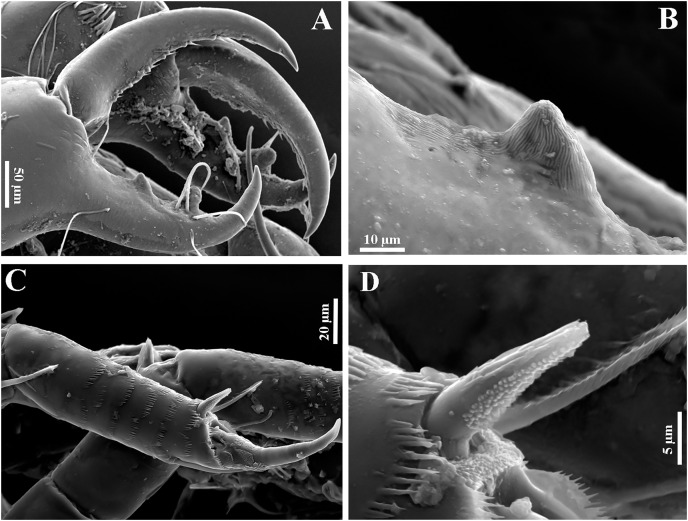
*Chondrochelia winfieldi* sp. nov. Topotype specimens. SEM images. Male, 2.3 mm, ECOSUR. (A) Dactylus and fixed finger of cheliped. (B) Proximal process on cutting edge of cheliped. (C) Scales on propodus of pereopod 3. (D) Detail of ventrodistal spine on propodus of pereopod 3. Photo credit: Luis Fernando Carrera-Parra.

**Type material**. Holotype ECOSUR 238, non-ovigerous female, 3.0 mm, Isla Verde, Veracruz, Mexico, 19°11′59.3″N 96°04′04.8″W, 1 m, coralline rock, October 27 2015. Paratypes: ECOSUR 239, adult male, 2.1 mm, same data as holotype. ECOSUR 240, five ovigerous females, 19 non-ovigerous females, three adult males, nine mancae, same data as holotype.

**Additional material**. CNCR 27142, two ovigerous females, six non-ovigerous females, Isla Verde, Veracruz, Mexico, in *Thalassia testudinium*, littoral, August 11 2011. CNCR 27144, 23 ovigerous females, 42 non-ovigerous females, 27 males, six mancae, Isla Verde, Veracruz, Mexico, littoral, in macroalgae, August 11 2011.

**Molecular material**. ECOSUR TANAI270-15, TANAI270-15, TANAI272-15, TANAI273-15, TANAI274-15 (one ovigerous female, one non-ovigerous female, one non-ovigerous female, one non-ovigerous female, one non-ovigerous female): Isla Verde, Veracruz, Mexico, 19°11′59.3″N 96°04′04.8″W, 1 m, coralline rock, October 27 2015.

**Diagnosis**. **Female**. *Mouthparts*. Left mandible with four denticled *pars incisiva*, *lacinia mobilis* smooth and pointed; maxilliped left basis with five plumose setae, right basis with four plumose setae. *Chelipeds*. Fixed finger cutting edge with three processes; dactylus cutting edge with three subproximal spinules. *Pereopods*. Pereopod 1 carpus with six distal plumose setae. Pereopod 2 carpus with three setae and two small setulate spines on distal extremity. Pereopod 4 propodus with three dorso-subdistal plumose setae. Pereopod 6 propodus with six dorso-subdistal plumose setae. **Male**. *Carapace* subrectangular. *Antennules*. Flagellum 1.5 times longer than peduncle article-1, with nine articles. *Chelipeds*. Fixed finger cutting edge with two processes, proximal shorter and pointed, distal longer, tubular and blunt. *Pereopods*. Pereopod 1 carpus with four plumose setae on distal extremity. Pereopod 3 merus with one slender setulate spine and one plumose seta on ventral side. Pereopod 4 propodus with three dorso-subdistal plumose setae. Pereopod 6 propodus with six dorso-subdistal plumose setae. **Both sexes**. *Uropods* with exopod biarticulate and endopod with five articles.

**Etymology**. This species name is after Dr. Ignacio Winfield in recognition of his contribution to the knowledge of the peracarid crustaceans of Mexico.

**Description. Non-ovigerous female**. Holotype ECOSUR 238, 3.0 mm (Fig. 14A). Body 6.4 times longer than broad. Carapace oval, 1.3 times longer than broad, 0.7 times as long as pereonites 1–3 together. Ocular lobes well defined, eyes pigmented. Pereon three times longer than carapace and 3.8 times longer than broad; all pereonites respectively, 2.3, 1.5, 1.5, 1.2, 1.5 and 2.3 times broader than long. Pleon 0.6 times as long as pereonites 1–3 together. Pleotelson about 0.3 times as long as pleon, posterior apex slightly projected, with four distal setae.

Antennule (Fig. 14B) with three long peduncular and one short flagellar articles. Article-1 about three times longer than broad, with two mesial setae, two subdistal simple setae, five sensory setae and scales. Article-2 about 0.3 times as long as peduncle article-1, 1.4 times longer than broad, with two distal simple setae and scales. Article-3 about 0.3 times as long as peduncle article-1, 2.5 times longer than broad, with two simple setae and one aesthetasc. Article-4 (= flagellum) small, with three simple setae.

Antenna (Fig. 14C) with six articles. Article-1 1.3 times longer than broad, narrow distally, naked. Article-2 1.3 times longer than broad, narrow proximally, with two slender distal spines. Article-3 longer than broad, with one slender distal spine. Article-4 3.4 times longer than broad, with three distal simple setae, one mesial and three distal sensory setae. Article-5 0.4 times as long as article-4, 2.3 times longer than broad, with two distal simple setae. Article-6 small, with three simple setae.

Labium and labrum setose as other leptocheliids (not illustrated).

Mandibles. *Pars molaris* well developed in both mandibles, with strong rugosity on masticatory surface. Left mandible (Fig. 14D) with four denticled *pars incisiva*; *lacinia mobilis* smooth, pointed. *Pars incisiva* of right mandible (Fig. 14E) bifid, distally crenulate, with two inner spinules.

Maxillule (Figs. 14F and 14G) with 10 robust spines distally and numerous setae laterally and on distal extremity.

Maxilliped (Figs. 14H–14J). Left basis with five distal plumose setae and right basis with four plumose setae on distal extremity. Palp article-1 about longer than endite, naked; article-2 largest, with one dorsal plumose seta and three ventrodistal plumose setae; article-3 with eight plumose setae ventrally; article-4 with eight ventro-subdistal plumose setae, two dorsal plumose setae, and scales. Endites with two plumose setae (one of them longer), three flat spines (two longer and one short), two coupling hooks, and setules on distal extremity.

Cheliped (Figs. 15A and 15B) basis 1.2 times longer than broad, with one small dorsodistal simple seta. Merus with three ventral setae. Carpus about twice as long as broad, with two dorsal-subproximal spinules and two dorso-subdistal setae, with three ventrodistal setae. Propodus with one dorsal seta near dactylus articulation, comb-row with five ventral setulate spines and scales. Fixed finger cutting edge with three processes, the first process prominent; with seven setae, three dorsal. Dactylus cutting edge with one ventroproximal seta, and three subproximal spinules.

Pereopod 1 (Fig. 15C) basis 3.7 times longer than broad, with one dorsoproximal plumose seta and one dorsoproximal sensory seta. Ischium with two ventral plumose setae. Merus with oblique articulation with carpus, 2.4 times longer than broad, with ventrodistal plumose seta. Carpus 0.7 times as long as merus, 1.7 times longer than broad, with six distal plumose setae. Propodus 0.6 times as long as merus and carpus together, 3.6 times longer than broad, with three dorso-subdistal plumose setae and one ventro-subdistal plumose seta. Dactylus with one dorsoproximal plumose seta and scales, about as long as carpus, 1.4 times longer than unguis, together with unguis as long as propodus.

Pereopod 2 (Fig. 15D) smaller than pereopod 1, basis 2.8 times longer than broad, with one dorsoproximal plumose seta and one dorsoproximal sensory seta. Ischium with one ventral plumose seta. Merus short, 1.7 times longer than broad, with one small ventrodistal setulate spine and one ventrodistal plumose seta. Carpus 0.8 times as long as merus, with three plumose setae and two small setulate spines on distal extremity. Propodus 0.5 times as long as basis, with three dorso-subdistal plumose setae and one small ventro-subdistal setulate spine. Dactylus with scales, 1.8 times longer than unguis, together with unguis 0.6 times as long as propodus.

Pereopod 3 (Fig. 15E) similar to pereopod 2, but propodus with two dorso-subdistal plumose setae and one ventro-subdistal spine.

Pereopod 4 (Fig. 15F) basis 1.9 times longer than broad, naked. Ischium with two ventral plumose setae. Merus with oblique articulation with carpus, 2.3 times longer than broad, with two small ventrodistal spines. Carpus 0.7 times as long as merus, with two plumose setae and three setulate spines on distal extremity. Propodus 0.5 times as long as merus and carpus together, with three dorsodistal plumose setae and two ventrodistal setulate spines. Dactylus with scales, 2.2 times longer than unguis, together with unguis 0.5 times as long as propodus.

Pereopod 5 (Fig. 15G) similar to pereopod 4, but propodus with two plumose setae and one pappose seta on distal extremity.

Pereopod 6 (Fig. 15H) similar to pereopods 4 and 5, but propodus with six dorso-subdistal plumose setae, two of them longer.

Pleopods similar to other members of genus *Chondrochelia* (not illustrated). Pleopod 1 peduncle with one ventrodistal circumplumose seta. Endopod with one middorsal circumplumose seta, one proximoventral circumplumose seta and 13 ventral plumose setae. Exopod with one proximoventral circumplumose seta and 23 ventral plumose setae.

Uropod (Fig. 15I) peduncle naked. Exopod 0.9 times as long as endopod article-1; biarticulate, article-1 small and with one subdistal simple seta with terminal pore, article-2 with two apical simple setae with terminal pore. Endopod with five articles; article-1 broader than other articles; articles 1 and 2 with two distal simple setae; article-3 shorter than other articles, with one sensory seta; articles 4 and 5 subequal; article-4 with one simple seta with terminal pore and one sensory seta; article-5 with five simple setae with terminal pore and one sensory seta.

**Adult male**. Paratype ECOSUR 240, 2.6 mm (Fig. 16A). Body 5.7 times longer than broad. Carapace subrectangular, 1.3 times longer than broad, 1.1 times longer than pereonites 1–3 together. Ocular lobes well defined, eyes pigmented. Pereon 2.2 times longer than carapace and three times longer than broad; all pereonites respectively, 4.0, 2.1, 2.1, 1.6, 1.6 and 2.5 times broader than long. Pleon 0.8 times as long as pereonites 1–3 together. Pleotelson 0.3 times as long as pleon, posterior apex with four simple setae and two sensory setae.

Antennule (Fig. 16B) 0.4 times as long as body. Article-1 0.5 times as long as carapace, 4.1 times longer than broad, with two small subproximal sensory setae and two distal simple setae. Article-2 about 0.5 times as long as peduncle article-1, 2.4 times longer than broad, with two distal simple setae and three sensory setae. Article-3 0.3 times as long as peduncle article-1, 1.7 times longer than broad, with two small distal simple setae and one sensory seta. Flagellum 1.5 times longer than peduncle article-1, with nine articles; each flagellar article with at least four aesthetascs; last article minute, with four distal simple setae.

Antenna (Fig. 16C) article-1 as long as broad, naked. Article-2 about 0.6 times as long as broad, with two slender distal spines. Article-3 0.7 times as long as broad, with one distal slender spine. Article-4 3.4 times longer than broad, with three distal simple setae, one mesial sensory seta, and one distal sensory seta. Article-5 six times longer than broad, with two distal simple setae. Article-6 small, with five distal simple setae.

Mouthparts reduced. Maxilliped rudimentary (not illustrated).

Cheliped (Figs. 16D and 16E) stout, 0.6 times as long as body. Basis 1.3 times longer than broad, with one dorso-subdistal seta. Merus wider distally, with three ventral simple setae. Carpus three times longer than broad, with three ventral simple setae, three dorso-subproximal simple setae, and one dorso-subdistal simple seta. Propodus with one simple seta near dactylus articulation, comb-row with 11 ventral setulate spines. Fixed finger cutting edge with two processes separated by a weak curvature; proximal process shorter and pointed; distal process longer, tubular and blunt; with seven simple setae, three dorsal. Dactylus cutting edge with one ventroproximal seta and nine spinules.

Pereopod 1 (Fig. 16F) basis 4.1 times longer than broad, with one dorsoproximal plumose seta. Ischium with one ventral plumose seta. Merus as long as carpus, with one ventrodistal plumose seta. Carpus 2.5 times longer than broad, with four plumose setae on distal extremity. Propodus 0.5 times as long as merus and carpus together, 3.3 times longer than broad, with five dorsodistal plumose setae and one ventro-subdistal slender spine. Dactylus with one subproximal plumose seta and scales, 2.3 times longer than unguis, together with unguis 0.8 times as long as propodus.

Pereopod 2 (Fig. 17A) basis four times longer than broad, with one dorso-subproximal seta and two dorso-subproximal sensory setae. Ischium with one ventral plumose seta. Merus as long as carpus, with one plumose seta and one setulate spine on ventrodistal margin. Carpus twice as long as broad, with three plumose setae and two setulate spines on distal extremity. Propodus about four times longer than broad, with two dorso-subdistal plumose setae and two ventro-subdistal setulate spines, with scales. Dactylus with one mesial plumose seta and scales, 0.5 times as long as carpus, 3.2 times longer than unguis, together with unguis 0.6 times as long as propodus.

Pereopod 3 (Fig. 17B) similar to pereopod 2, but propodus with one ventro-subdistal setulate spine.

Pereopod 4 (Fig. 17C) with broad basis, with one dorso-subproximal plumose seta and two dorso-subproximal sensory setae. Ischium with two ventral plumose setae. Merus as long as carpus, with two ventrodistal setulate spines and scales. Carpus 2.3 times longer than broad, with two plumose setae and three setulate spines on distal extremity. Propodus 4.5 times longer than broad, with three dorso-subdistal plumose setae and two ventro-subdistal slender setulate spines, with scales. Dactylus with scales, 0.4 times as long as carpus, 2.5 times longer than unguis, together with unguis 0.5 times as long as propodus.

Pereopod 5 (Fig. 17D) similar to pereopod 4, but basis with one midventral plumose seta, one ventro-subdistal sensory seta, and two dorso-proximal sensory setae. Propodus with four dorsodistal plumose setae and two ventro-subdistal slender setulate spines.

Pereopod 6 (Fig. 17E) similar to pereopods 4 and 5, but propodus with six dorso-subdistal plumose setae and two ventro-subdistal slender setulate spines.

Pleopods similar to other members of genus *Chondrochelia* (not illustrated). Peduncle with one ventrodistal circumplumose seta. Endopod with one middorsal circumplumose seta, one proximoventral circumplumose seta and 14 ventral plumose setae. Exopod with one proximoventral circumplumose seta and 23 ventral plumose setae.

Uropod (Fig. 17F) peduncle with six dorso-subdistal setae. Exopod 1.2 times longer than endopod article-1; biarticulate, article-1 shorter than article-2, article-1 with one distal simple seta with terminal pore, article-2 with two simple setae with terminal pore. Endopod with five articles; article-1 broader than the following articles, with two simple setae with terminal pore; article-2 with three simple setae with terminal pore and one sensory seta; article-3 shorter than other articles, with three simple setae with terminal pore; article-4 longer than other articles, with two simple setae with terminal pore; article-5 more slender than other articles, with five simple setae with terminal pore.

The SEM analysis allowed us to observe that females have simple setae and setules on the antennules (Fig. 18A), numerous scales on the base of the maxilliped (Fig. 18B), the detail of process on the cutting edge of the cheliped (Fig. 18C), the plumose setae, scales, setulate spines and pappose seta on the carpus and propodus of the pereopods (Figs. 18D–18F). In males, both processes on the cutting edge of the fixed finger of cheliped have striated surfaces (Figs. 19A and 19B); also, the spines on the propodus of pereopods 2–6 are ornamented with several small protuberances (Figs. 19C and 19D).

**Variability**. Non-ovigerous female, CNCR 27142, 2.0 mm. Uropod endopod with four articles. Male, CNCR 27144, 1.7 mm. Antennule flagellum with seven articles. Cheliped fixed finger cutting edge with seven simple setae. Uropod endopod with four articles. Male, CNCR 27144, 2.3 mm. Antennule flagellum with eight articles. Cheliped propodus comb-row with14 ventral setulate spines. Cheliped fixed finger cutting edge with nine setae. Cheliped dactylus cutting edge with 10 spinules.

**Distribution**. Know only from type locality.

**Type locality**. Isla Verde, Veracruz, Mexico.

**Remarks**. *Chondrochelia winfieldi* sp. nov. resembles *C. algicola* (Harger, 1878) from Massachusetts, USA, because the adult females of both species have an oval carapace, the *pars incisiva* of the right mandible is crenulate and with bifurcated apex, and by having the maxilliped endites with two long and one short, flattened setae. However, they have evident differences with females of *C. winfieldi* sp. nov. having the left mandible with a four denticled *pars incisiva* and pointed *lacinia mobilis*, while in *C. algicola* these are five denticled and stout respectively; *C. winfieldi* sp. nov. has three processes on the cheliped fixed finger cutting edge, whereas *C. algicola* has five; *C. winfieldi* sp. nov. has three spinules on the cutting edge of the cheliped dactylus, while in *C. algicola* it is smooth; *C. winfieldi* sp. nov. has six distal setae on the pereopod 1 carpus, but four in *C. algicola*; *C. winfieldi* sp. nov. has a biarticulate uropod exopod, uniarticulate in *C. algicola*; *C. winfieldi* sp. nov. has a uropod endopod with five articles, but six in *C. algicola*.

The males of both species have large eyes, the carapace is as long as broad, and it is as long as pereonites 1–3, antenna article-1 is naked; the cheliped fixed finger cutting edge has a proximal process and a mesial process, and they have two simple setae on the pereopod 3 propodus. They can be distinguished because in *C. winfieldi* sp. nov. the antennule length is less than 0.5 times the body length, whereas in *C. algicola* is more than 0.5 times; in *C. winfieldi* sp. nov. the proximal process on the cutting edge is shorter, while in *C. algicola* is longer; in *C. winfieldi* sp. nov. the distal process on cutting edge is longer, tubular and blunt, whereas in *C. algicola* it is shorter and apically crenulate. Furthermore, *C. winfieldi* sp. nov. has two ventro-subdistal setulate spines on the pereopod 2 propodus, while *C. algicola* has one ventro-subdistal slender spine; in *C. winfieldi* sp. nov. the uropod has a biarticulate exopod and endopod with five articles, whereas *C. algicola* has a uniarticulate exopod and endopod with six articles.

The females of *C. caribensis* sp. nov. and *C. winfieldi*, sp. nov., as well as other members of the genus *Chondrochelia*, are similar because they have an ovate carapace, antennule with five articles, maxilliped endites with long lateral seta and three flat spines (generally), the uropod exopod with one or two articles, and the uropod endopod with five articles (mostly). However, they differ because *C. caribensis* sp. nov. has four outer denticles and inner setules in the *lacinia mobilis* of the left mandible, while in *C. winfieldi* sp. nov. the *lacinia mobilis* is smooth and pointed. Also, *C. caribensis* sp. nov. has the cutting edge of dactylus crenulate proximally and four distal processes, whereas *C. winfieldi* sp. nov. has only three subproximal spinules on the cutting edge; in *C. caribensis* sp. nov. the propodus of pereopod 4 has five plumose setae and one pappose seta on the distal extremity, while *C. winfieldi* sp. nov. has three plumose dorso-subdistal setae; also, *C. caribensis* sp. nov. has 10 plumose dorso-subdistal setae on propodus of pereopod 6, whereas *C. winfieldi* sp. nov. has only six.

Regarding males, these species differ because *C. caribensis* sp. nov. has a round carapace, while in *C. winfieldi* sp. nov. it is subrectangular; in *C. caribensis* sp. nov. the antennule flagellum is as long as peduncle article-1, whereas in *C. winfieldi* sp. nov. the flagellum is 1.5 times longer. Furthermore, *C. caribensis* sp. nov. has six distal plumose setae on the pereopod 1 carpus, while *C. winfieldi* sp. nov. has four plumose setae; *C. caribensis* sp. nov. has eight plumose dorso-subdistal setae on the pereopod 6 propodus, whereas *C. winfieldi* sp. nov. has six.

Females of *C. winfieldi* sp. nov., and *C. dubia* differ mainly because in *C. dubia* the third peduncular article of the antennule has a length-width ratio of 4.2, while in *C. winfieldi* sp. nov. it is 2.5; in *C. dubia* the values of the length-width ratio of articles 4 and 5 are 5.0 and 4.5, while in *C. winfieldi* sp. nov. are 3.4 and 2.3. Furthermore, in *C. dubia* the uropod is almost 0.16 times as long as the body, whereas in *C. winfieldi* sp. nov. it is less than 0.16 times as long; *C. dubia* has an uniarticulate uropod exopod, while in *C. winfieldi* sp. nov. it is biarticulate. Other differences are shown in Table 2.

### *Chondrochelia mexicana* (Jarquín-González, García-Madrigal & Carrera-Parra, 2015)

Figures 20–21

**Figure 20:**
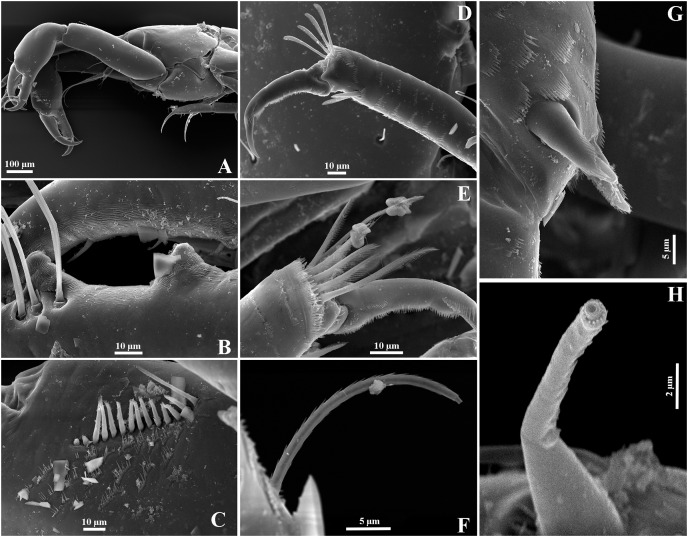
*Chondrochelia mexicana* male from Estacahuite, Oaxaca, Mexico. SEM images. Male, 2.1 mm, ECOSUR. (A) Chelipeds. (B) Processes on cutting edge of cheliped. (C) Detail of comb-row of propodus of cheliped. (D) Detail of the ornamentation of the propodus of pereopod 4. (E) Detail of the ornamentation of the propodus of pereopod 6. (F) Plumose seta with terminal pore on propodus of pereopod 6. (G) Setulate spines and scales on merus of pereopod 4. (H) Detail of spine on article-2 of uropodal endopod. Photo credit: Luis Fernando Carrera-Parra.

**Figure 21:**
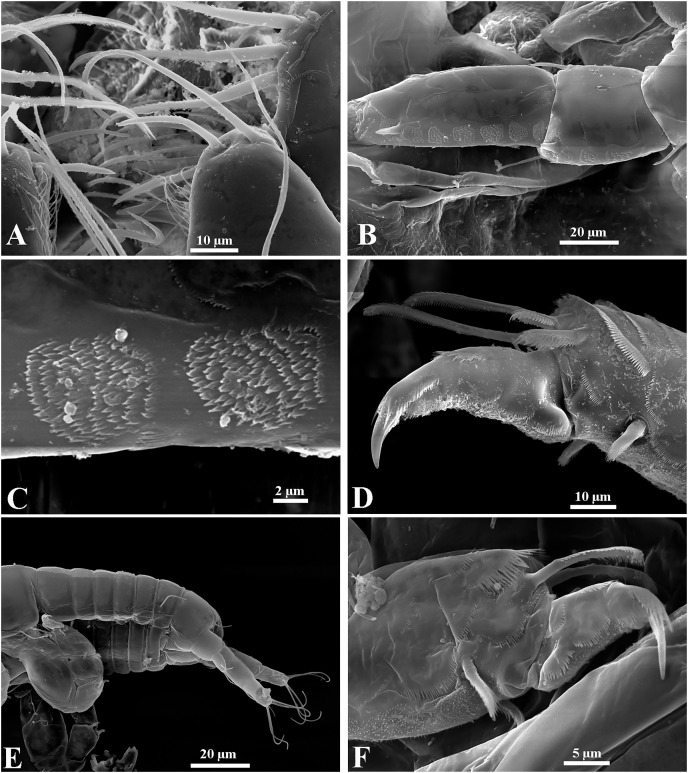
*Chondrochelia mexicana* female and manca from Estacahuite, Oaxaca, Mexico. SEM images. Non-ovigerous female, 2.0 mm, ECOSUR. (A) Plumose setae on palp article-4 of maxilliped. (B–C) Detail of scales on carpus and propodus of pereopod 2. (D) Plumose and pappose setae on propodus of pereopod 3. Manca, 0.6 mm, ECOSUR. (E) Pereopods 6 and uropods. (F) Plumose setae and scales on propodus of pereopod 6. Photo credit: Luis Fernando Carrera-Parra.

*Leptochelia mexicana Jarquín-González, García-Madrigal & Carrera-Parra (2015)*: 510–516, figs. 2–5.

*Chondrochelia mexicana*: Guţu & Bird, 2017: 591.

**Type material**. Holotype, male, UMAR-Pera 015, Playa Coral, Isla Ixtapa, Guerrero, Mexico, rocks in tide pool, littoral, September 19 2007.

**Additional material**. ECOSUR-C1086 two ovigerous females, 27 non-ovigerous females, five adult males, 3 mancae, Playa Estacahuite, Oaxaca, Mexico, 15°40′5.16″N 96°28′54.84″W, 2 m, in coralline rock, November 04 2015. ECOSUR-C1087 20 non-ovigerous females, La Boquilla, Oaxaca, Mexico, 15°40′57.4″N 96°27′54.0″W, 3 m, in coralline rock, November 05 2015. ECOSUR-C1088 three ovigerous females, 38 non-ovigerous females, six mancae, La Boquilla, Oaxaca, Mexico, 15°40′57.4″N 96°27′54.0″W, 4 m, in brown algae *Padina* sp., November 05 2015.

**Molecular material**. ECOSUR TANAI223-15 (one adult male): La Boquilla, Oaxaca, Mexico, 15°40′57.4″N 96°27′54.0″W, 3 m, in coralline rock, November 05 2015. ECOSUR TANAI238-15, ECOSUR TANAI241-15, ECOSUR TANAI243-15, (one non-ovigerous female, one juvenile female, one juvenile female): La Boquilla, Oaxaca, Mexico, 15°40′57.4″N 96°27′54.0″W, 4 m, in brown algae *Padina* sp., November 05 2015. ECOSUR TANAI263-15, ECOSUR TANAI264-15, ECOSUR TANAI267-15 (one adult male, one non-ovigerous female, one manca): Playa Manzanillo, Guerrero, Mexico, 16°50′26.5″N 99°54′35.6″W, 2.5 m, in sponge, November 10 2015.

**Remarks**. Using a SEM, we were able to observe that the antennules and chelipeds, described and illustrated by Jarquín-González, García-Madrigal & Carrera-Parra (2015, Figs. 2–5) are the only structures having true simple setae (without terminal pore or ornamentation), except in the comb-row of the cheliped propodus where there are ventral setulate spines (Figs. 20A and 20C). The pereopods are usually the most ornate structures because all have sensory setae, plumose setae with a terminal pore, setulate spines, and scales (Figs. 20D, 20E and 20G). Also, all uropod setae are plumose with a terminal pore (Fig. 20F).

Regarding males, chelipeds have striated surface on both processes on the cutting edge (Fig. 20B) and they have three proximal processes on the cutting edge of the dactylus; furthermore, the spiniform seta present on the uropod endopod article-2 has a thin projection with a distal pore surrounded by several denticles (Fig. 20H). In the females, all the mouthparts have scales and plumose setae as in the maxilliped (Fig. 21A). The pereopods have complex scales, plumose and pappose setae, and setulate spines (Figs. 21B–21D). Also, although the anatomical structures are partially developed in mancae, the cuticular ornamentation is already present (Figs. 21E and 21F).

**Distribution**. Southern Mexican Pacific. From Playa Coral, Isla Ixtapa, Guerrero to La Boquilla, Oaxaca, Mexico.

### *Chondrochelia ortizi* (Jarquín-González, 2016)

*Leptochelia ortizi Jarquín-González, 2016*: 395–404, figs. 1–5.

*Chondrochelia ortizi*: Guţu & Bird, 2017: 591.

**Type material**. Holotype, male, ANC 07.1.4.002, Punta del Este, 21°33′29.41″N 82°32′55.20″W, Isla de la Juventud, Cuba, station 4, rock bottom, November 02 1979. Paratype: ECOSUR 234 one male from Punta del Este, Isla de la Juventud, Cuba, 21°33′31.70″N 82°33′10.32″W, station 3, between coral and *Thalassia testudinum*, 6–8 m, November 04 1979.

**Additional material**. ECOSUR-C1072 four ovigerous females, four non-ovigerous females, one manca, Gulf of Guanahacabibes, Pinar del Rio, Cuba, 21°53′23.9″N 84°48′47.0″W, station 15, 0.5 m, in mangrove sediment, June 10 2014. ECOSUR-C1073 two non-ovigerous females, one manca, Gulf of Guanahacabibes, Pinar del Rio, Cuba, 22°00′34.9″N 84°48′47.0″W, station 14, 5 m, in seagrass, June 10 2014. ECOSUR-C1089 six ovigerous females, 79 non-ovigerous females, two males, 25 mancae, Champotón, Campeche, Mexico, in marine grass *Halodule* sp., 4 m, June 17 2014.

**Molecular material**. ECOSUR TANAI019-15 (one adult male): Champotón, Campeche, Mexico, 19°20′20.4″N 90°44′20.4″W, 4 m, in marine grass *Halodule* sp., June 17 2014. ECOSUR TANAI089-15 (one ovigerous female): Gulf of Guanahacabibes, Cuba, 21°53′24.0″N 84°48′46.8″W, in mangrove, June 10 2014.

**Remarks**. According to Jarquín-González (2016), the males of *Chondrochelia ortizi* are characterized by having a subrectangular carapace, an antennule flagellum with eight articles, cheliped fixed finger cutting edge with two small and unequal mesial processes, cheliped dactylus cutting edge with six spinules, pereopod 1 carpus with six distal setae, and pereopod 6 propodus with five distal setae. Whereas the females have the antennule article-1 3.6 times longer than broad, the left mandible *lacinia mobilis* with fine inner setules, and the cheliped dactylus cutting edge with three proximal spinules and seven processes. In both sexes, the uropod endopod has five articles.

This species was only known from the Gulf of Guanahacabibes and Isla de la Juventud, Cuba. However, from the morphological and molecular analysis, we were able to confirm that this species is also distributed in waters of the Gulf of Mexico, specifically at Champotón, Campeche, Mexico.

**Distribution**. Gulf of Mexico (Champotón, Campeche, Mexico) and Cuba (Gulf of Guanahacabibes and Isla de la Juventud).

### Molecular analysis

**Figure 22:**
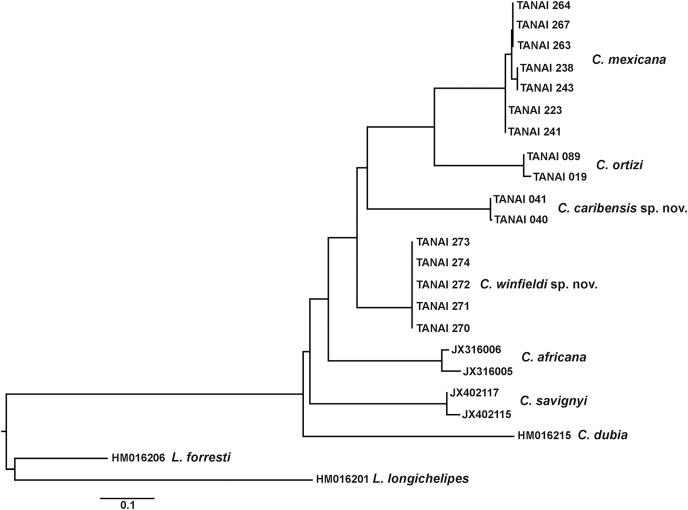
Maximum likelihood tree of COI sequences. Using Hasegawa–Kishino–Yano with a discrete Gamma distribution with five rate categories and by assuming that a certain fraction of sites is evolutionarily invariable (HKY+G+I).

The molecular analyses based on the COI gene supported the morphological differences found between the species in this study (Fig. 22). *Chondrochelia caribensis* sp. nov. has a 23% genetic divergence (K2P) from *C. winfieldi* sp. nov., and both new species are close related to *C. ortizi* and *C. mexicana*, with genetic divergences ranging from 27.7% to 24.2%. Compared to *C. dubia*, the species name previously given to the specimens of both new species, *C. caribensis* sp. nov. and *C. winfieldi* sp. nov. have a 34.7% and 34.4% genetic divergence, respectively. These genetic divergences clearly exceed the lowest values (4.9%) of interspecific divergence found in crustaceans (Costa et al., 2007; Montiel-Martínez et al., 2008). Unfortunately, the only available sequence identified as *C. dubia* was obtained from a specimen collected distant from its type locality, so it is quite possible that it does not belong to *C. dubia*. However, the result obtained highlights the existence of pseudo-cryptic species under the name *C. dubia* in the Caribbean and Gulf of Mexico region. To reinforce our hypothesis, the redescription of *C. dubia* based on morphological and molecular data of topotypical specimens is essential; unfortunately, we could not obtain these. Our results, both morphological and molecular, nevertheless establish that *C. dubia* is not distributed in the Mexican Caribbean and the Gulf of Mexico, as had previously been proposed. Also, we found that the species *C. ortizi*, previously only found in Cuba, is also present in the Gulf of Mexico.

## Discussion

Analysis of material from the United States, Cuba and Mexico, has resulted in six species of the genus *Chondrochelia* being recorded, two of them described in this paper.

In the last decade, the traditional taxonomic descriptions have been strengthened by using molecular markers, especially COI. For tanaidaceans, the use of integrative taxonomy using morphological and molecular data has been crucial to clarify problems related to cryptic or pseudo-cryptic species, polymorphism and ontogeny, and to establish general patterns of distribution. These demonstrate that the wide distributions of some species are questionable and deserve to be corroborated. This approach has also shown that there is an underestimation in the species diversity.

Here, we show a clear example of this issue; after the reexamination of specimens of “*Leptochelia dubia*” using a morphological and molecular information, we found two new species for science, *Chondrochelia caribensis* sp. nov. and *Chondrochelia winfieldi* sp. nov. that represent the first *Chondrochelia* species described for the Mexican Caribbean and Gulf of Mexico, respectively. Also, we were able to establish an increase in the distribution range for *Chondrochelia mexicana* and *Chondrochelia ortizi*.

The use of SEM revealed that the species have a more complex morphology and ornamentation than previously described for the genus; even in mancae. According to Zimmer, Araujo & Bond-Buckup, 2009 this taxonomic tool is important because contributes significantly to the morphological comparison at the levels of species, genera and families.

## Conclusions

*Chondrochelia* from America included four species, with *C. dubia* thought to have a wide distribution. Here, we described two new species using morphological and molecular data, and another poorly characterized species was redescribed; also, the distribution of two species was expanded within their geographic regions where they were previously described. In contrast, the supposed distribution of *C. dubia* in the Mexican Caribbean and the Gulf of Mexico is rejected. Using morphological and molecular data offers a good strategy for the study of the biological diversity of small crustaceans since it allows reliable species discrimination and strengthens the taxonomic identification that in these groups is usually quite complex due to their size, crypticity and biology. It is important to continue advancing the knowledge of tanaidaceans, especially in regions with high biological diversity such as the Mexican Caribbean. Determining the current taxonomic status of the species will make it possible to deepen in the ecological knowledge of the group and thus establish lines of research that reinforce the conservation and management strategies of marine and coastal natural resources.

## Supplemental Information

10.7717/peerj.12773/supp-1 Supplemental Information 1 COI sequences *Chondrochelia caribensis* sp. nov.

10.7717/peerj.12773/supp-2 Supplemental Information 2 COI sequences *Chondrochelia winfieldi* sp. nov.

10.7717/peerj.12773/supp-3 Supplemental Information 3 COI sequences *Chondrochelia ortizi*.

10.7717/peerj.12773/supp-4 Supplemental Information 4 COI sequences *Chondrochelia mexicana*.

## Additional Information and Declarations

### Data Availability

The following information was supplied regarding data availability: The COI sequences of *Chondrochelia caribensis* sp. nov. (TANAI040-15, TANAI041-15), *Chondrochelia winfieldi* sp. nov. (TANAI270-15, TANAI271-15, TANAI272-15, TANAI273-15, TANAI274-15), *Chondrochelia mexicana* (TANAI223-15, TANAI238-15, TANAI241-15, TANAI243-15, TANAI263-15, TANAI264-15, TANAI267-15), and *Chondrochelia ortizi* (TANAI019-15, TANAI089-15) are available within the project Tanaidacea from Mexico (TANME) at Barcode of Life Data System. *C. caribensis* sp. nov. TANAI040-15
https://www.boldsystems.org/index.php/Public_RecordView?processid=TANAI040-15
TANAI041-15
https://www.boldsystems.org/index.php/Public_RecordView?processid=TANAI041-15
*C. winfieldi* sp. nov. TANAI270-15
https://www.boldsystems.org/index.php/Public_RecordView?processid=TANAI270-15
TANAI271-15
https://www.boldsystems.org/index.php/Public_RecordView?processid=TANAI271-15
TANAI272-15
https://www.boldsystems.org/index.php/Public_RecordView?processid=TANAI272-15
TANAI273-15
https://www.boldsystems.org/index.php/Public_RecordView?processid=TANAI273-15
TANAI274-15
https://www.boldsystems.org/index.php/Public_RecordView?processid=TANAI274-15

*C. ortizi*
TANAI019-15
https://www.boldsystems.org/index.php/Public_RecordView?processid=TANAI019-15
TANAI089-15
https://www.boldsystems.org/index.php/Public_RecordView?processid=TANAI089-15

*C. mexicana*
TANAI223-15
https://www.boldsystems.org/index.php/Public_RecordView?processid=TANAI223-15
TANAI238-15
https://www.boldsystems.org/index.php/Public_RecordView?processid=TANAI238-15
TANAI241-15
https://www.boldsystems.org/index.php/Public_RecordView?processid=TANAI241-15
TANAI243-15
https://www.boldsystems.org/index.php/Public_RecordView?processid=TANAI243-15
TANAI263-15
https://www.boldsystems.org/index.php/Public_RecordView?processid=TANAI263-15
TANAI264-15
https://www.boldsystems.org/index.php/Public_RecordView?processid=TANAI264-15
TANAI267-15
https://www.boldsystems.org/index.php/Public_RecordView?processid=TANAI267-15

### New Species Registration

The following information was supplied regarding the registration of a newly described species: Publication LSID: urn:lsid:zoobank.org:pub:DB236268-1523-41AF-8701-16A3E1D696F0. *Chondrochelia caribensis* LSID: urn:lsid:zoobank.org:act:45FD18D0-1D0F-4921-98DD-3154A13460A7. *Chondrochelia winfieldi* LSID: urn:lsid:zoobank.org:act:E8BD8518-865F-49AE-90AC-E2F95CD121AF.
